# DNA Damage Recognition by Bacterial and Human Adenine-DNA Glycosylases: Insights from Non-Canonical Substrates

**DOI:** 10.3390/biom16081214

**Published:** 2026-08-20

**Authors:** Ulan Sarsenbayeva, Didier Gasparutto, Nicolas Geacintov, Alexander A. Ishchenko, Gulzhan Zhamanbayeva, Kamalidin O. Sharipov, Carlos H. Trasviña-Arenas, Sheila S. David, Dmitry O. Zharkov, Bakhyt T. Matkarimov, Murat Saparbaev, Sabira Taipakova

**Affiliations:** 1 Faculty of Biology and Biotechnology, Al-Farabi Kazakh National University, Almaty 050040, Kazakhstan; ulansarsenbayeva@gmail.com (U.S.); gulzhan.kaznu.kz@gmail.com (G.Z.); 2 Department of Biochemistry, Asfendiyarov Kazakh National Medical University, Almaty 050012, Kazakhstan; sharipov.k@kaznmu.kz; 3 CEA, CNRS, Institut National Polytechnique de Grenoble, Institut de Recherche Interdisciplinaire de Grenoble, Systèmes Moléculaires et nanoMatériaux pour l’Énergie et la Santé—Unité Mixte de Recherche 5819, University Grenoble Alpes, 38000 Grenoble, France; didier.gasparutto@cea.fr; 4 Chemistry Department, New York University, 31 Washington Place, New York, NY 10003, USA; ng1@nyu.edu; 5 CNRS-UMR9019 Genome Integrity and Cancers, Gustave Roussy Cancer Campus, Université Paris-Saclay, 94805 Villejuif, France; alexander.ishchenko@gustaveroussy.fr; 6 Structural and Functional Genomics Laboratory, M.A. Aitkhozhin Institute of Molecular Biology and Biochemistry, Almaty 050012, Kazakhstan; 7 Centro de Investigación sobre el Envejecimiento, Centro de Investigación y de Estudios Avanzados (CINVESTAV), Unidad Sede Sur, Tlalpan, Ciudad de México 14330, Mexico; carlos.trasvina@cinvestav.mx; 8 Chemical Biology Graduate Program, Department of Chemistry and Chemistry, University of California, Davis, CA 95616, USA; ssdavid@ucdavis.edu; 9 SB RAS Institute of Chemical Biology and Fundamental Medicine, Lavrentiev Av. 8, Novosibirsk 630090, Russia; oxoguanine@gmail.com; 10 Department of Natural Sciences, Novosibirsk State University, 2 Pirogova St., Novosibirsk 630090, Russia; 11 National Laboratory Astana, Nazarbayev University, Astana 010000, Kazakhstan; bmatkarimov@nu.edu.kz; 12 L.N. Gumilyov Eurasian National University, Astana 010000, Kazakhstan; 13 Scientific Research Institute of Biology and Biotechnology Problems, Al-Farabi Kazakh National University, Almaty 050040, Kazakhstan

**Keywords:** adenine-DNA glycosylase, base excision repair, cyclobutane pyrimidine dimer, (6,4)photoproduct, 8,5′-*cyclo*-2′-deoxyadenosine, 1,3-d(GpNpG) cisplatin intra-strand crosslink, ethenobases, apurinic/apyrimidinic site, 7,8-dihydro-8-oxoguanine, 1,2-dihydro-2-oxoadenine, aberrant DNA repair

## Abstract

The *Escherichia coli* adenine-DNA glycosylase (MutY) and its human homologue, MUTYH, protect cells against oxygen-free radical-induced mutagenesis by excising regular adenine impaired with 8-oxo-7,8-dihydro-guanine (8oxoG) in the base excision repair (BER) pathway. However, removal of adenine by MutY and MUTYH from an A·8oxoG pair generated via misincorporation of an oxidized nucleotide during DNA synthesis might induce A·T→C·G transversions. Here, to examine MutY and MUTYH in vitro activities, we used short synthetic DNA duplexes in which the target adenine residue was positioned opposite a variety of DNA base modifications. MUTYH does not excise mismatched adenine in non-canonical DNA substrates, whereas MutY excises adenine mispaired with 1,3-d(GpNpG) cisplatin intra-strand crosslink. In addition, we characterized four MUTYH variants associated with cancer risk, which exhibit the following order of DNA glycosylase deficiency: WT ≥ G169D > G202E ≈ Y165C >> D222N. Human adenine-DNA glycosylase MUTYH and its mutant variants, contrary to bacterial MutY, are not prone to aberrant removal of regular adenine residues opposite modified residues in DNA duplexes. We hypothesize that *E. coli* MutY is prone to aberrant repair under certain conditions and that this may prevent incorporation of adenine opposite blocking lesions in the template strand.

## 1. Introduction

The majority of DNA base modifications generated via oxidation, alkylation and spontaneous deamination are removed in the base excision repair (BER) pathway [1]. BER is initiated by a DNA glycosylase, which excises the abnormal base by cleaving the *N*-glycosidic bond, generating either an apurinic/apyrimidinic (AP) site or a single-stranded break in DNA. Hydroxyl radicals (OH·) react preferentially with the C8 carbon of guanine in DNA to generate 7,8-dihydro-8-oxoguanine (8oxoG) and, in the nucleotide pool, to produce 8-oxo-2′-deoxyguanosine-5′-triphosphate (8oxodGTP). 8oxoG is a major, highly mutagenic oxidative DNA lesion in aerobic organisms, which induces G·C→T·A transversions if not eliminated [2]. To counteract the mutagenic effect of 8oxoG, cells have evolved numerous overlapping DNA excision repair and damage tolerance pathways. In *E. coli*, the GO pathway comprises three enzymes: MutT, which hydrolyzes 8oxodGTP to cleanse the cellular dNTP pool, thereby preventing its incorporation into DNA [3]; the bifunctional Fapy-DNA glycosylase/AP lyase Fpg/MutM, which excises the 8oxoG opposite cytosine in duplex DNA [4]; and, finally, the monofunctional adenine-DNA glycosylase MutY/MicA, which excises adenine when it is misincorporated opposite unrepaired 8oxoG in the DNA template [5]. Combined results from structural, biochemical and cellular studies have shown that MutY enzymes catalyze N-glycosidic bond cleavage using a double-displacement mechanism that relies on a nucleophilic catalytic residue Asp and the protonation of the adenine base by a catalytic Glu residue [6,7,8]. Multiple lines of evidence establish that the GO pathway protects the genome against mutations induced by guanine oxidation in DNA and the nucleotide pool across various species [9,10]. For instance, the *E. coli fpg mutY* double mutant displays an extreme mutator phenotype with a 10,000-fold increase in the rate of G·C→T·A transversions, which can be partly reversed by overexpression of the *Fpg* gene [11]. In parallel, the *E. coli mutT* strain also exhibits a marked mutator phenotype, characterized by strict mutational specificity: only A·T→C·G transversions are increased [12]. Interestingly, the introduction of a *mutY* mutation reduces the frequency of spontaneous A·T→C·G transversions in both *E. coli mutT* and wild-type (WT) strains; these observations suggest that MutY may participate in the error-prone post-replication repair pathway by removing the correct adenine from the DNA template strand when it is positioned opposite a misincorporated 8-oxo-dGTP residue [13].

MUTYH (MYH) is a human structural and functional homologue of the *E. coli* MutY protein, which exhibits DNA substrate specificities similar to those of its bacterial counterpart. Human and murine cell lines harboring biallelic mutations in the *MUTYH* gene exhibit a spontaneous mutator phenotype [14]; pathogenic germline variants of the MUTYH gene are associated with MUTYH-associated polyposis (MAP), a condition classified among certain types of familial colorectal tumors lacking germline mutations in the APC gene [15]. During DNA replication, MUTYH could be directed to the newly synthesized DNA strand containing an adenine mispaired with 8-oxoG through multiple specific protein–protein interactions [16,17,18]. These interactions tightly control the excision of normal adenines in order to minimize the aberrant excision of the adenine on the template strand when it is paired with a misincorporated 8-oxodGTP [19].

Mammalian MUTYH has a few counterintuitive properties, which make it different from other DNA glycosylases (reviewed in [20]). Biallelic germline pathogenic variants in MMR genes are associated with a predisposition to a colorectal cancer syndrome characterized by microsatellite instability (MSI) [21,22]. In contrast, the majority of MAP tumors are microsatellite stable [23], suggesting a genetic interaction between MUTYH and MMR [24]. Indeed, it was shown that double knockout Mutyh^-/-^ Msh2^-/-^ mice have a two-fold increased median lifespan compared to single Msh2-KO mice due to a strong delay in B cell lymphoma formation, suggesting that the cancer susceptibility conditions of Msh2^-/-^ mice depend in large part on MUTYH activity [25].

Furthermore, several studies have shown that, in cells lacking certain DNA repair pathways, MUTYH contributes to increased sensitivity to genotoxic stress. For example, synthetic lethality in colorectal cancer cell lines caused by either MSH2 and DNA polymerase β or MLH1 and DNA polymerase γ deficiencies can be rescued by MUTYH knockdown [26], suggesting that MUTYH can induce cell death in repair-deficient cells [27,28]. It was shown that reduced expression of MUTYH in human and mouse cells leads to increased resistance to alkylating agents [29]. Surprisingly, MUTYH played a more important role in cellular resistance than genes associated with the repair of alkylation DNA damage, such as the DNA methyltransferase MGMT and the mismatch repair enzymes MSH2 and MSH6 [30]. More recently, Mazouzi and colleagues demonstrated that the sensitivity of nucleotide excision repair (NER) pathway-deficient cells to UV light could be partially suppressed by the loss of the MUTYH gene [31]. The authors suggested that the MUTYH protein might interfere with the alternative NER-independent removal of UV-induced pyrimidine dimers in XPA-deficient cells.

In general, UV radiation generates two types of DNA lesions: the cyclobutane pyrimidine dimer (CPD) (~75%) and the pyrimidine (6–4) pyrimidone photoproduct [(6–4) photoproduct; 6–4PP] (~20%). Both photoproducts strongly inhibit DNA replication and transcription, leading to cytotoxicity and mutagenesis, with CPDs occurring more frequently than 6–4PPs [32]. UV mutagenesis is characterized by a specific mutational profile, marked by a very high frequency of C→T transitions at dipyrimidine sites in DNA; it has been shown that the rate of cytosine deamination is higher within CPDs than in undamaged DNA, which could explain the observed mutational signatures [33]. Previously, we showed that bacterial MutY does not excise regular adenine residues opposite UV-induced pyrimidine dimers and is not involved in UV-induced mutagenesis in *E. coli* strains tested [34]. However, a plausible hypothesis could be that the severe clinical manifestations in patients with Xeroderma pigmentosum (XP) may result from the aberrant excision, catalyzed by MUTYH, of normal adenines opposite UV-induced pyrimidine dimers in human cells, leading to futile repair and extreme genotoxic stress within the irradiated cells.

The recognition of regular T opposite damaged adenine by TDG, described in our earlier study [35], suggests that mismatch-specific MutY and MUTYH glycosylases might also excise normal A opposite residues other than 8-oxoG and guanine. To further investigate the potentially aberrant activities of mismatch-specific DNA glycosylases, in this study we characterized in vitro the activities of purified *E. coli* MutY and human MUTYH proteins on short synthetic oligonucleotide duplexes containing various nucleobase modifications, including both bulky and non-bulky adducts. In addition, we characterized mutant variants of MUTYH associated with cancer risk towards classical and non-canonical DNA substrates to examine whether missense mutations may influence DNA substrate specificity. The role of MutY and MUTYH glycosylase activities on non-canonical DNA substrates in DNA damage-induced mutagenesis is discussed.

## 2. Materials and Methods

### 2.1. Strains and Proteins

The *E. coli* Arctic BL21 (DE3) cells were purchased from Novagen-EMD4Biosciences (Merck Chemicals, Nottingham, UK). Phage T4 polynucleotide kinase was purchased from New England Biolabs (Evry, France). The purified BER proteins, including major human AP endonuclease 1 (APE1) and a truncated version of human uracil-DNA glycosylase (hUNGΔ84), were from laboratory stocks [36]. The expression and purification of AlkA, MutY and MUTYH are described below. The enzymatic activities of DNA repair proteins were checked using their classical DNA substrates just prior to use.

Site-directed mutations D222N, Y165C, G169D and G202E within the MUTYH coding sequence in pET28b-MBP-MUTYH were generated by the QuikChange site-directed mutagenesis kit according to the manufacturer’s instructions (Stratagene Europe, Amsterdam, The Netherlands). Oligonucleotide primers used to generate MBP-MUTYH-D222N, MBP-MUTYH-Y165C, MBP-MUTYH-G169D, and MBP-MUTYH-G202E are shown in Table 1.

### 2.2. Oligodeoxyribonucleotides

Sequences of the DNA oligonucleotides used in this work are shown in Table 1. All regular oligonucleotides and oligonucleotides containing base modifications, such as U, THF, 8oxoG, 5ohC, αdA, εC, εA and cdA, were synthesized by Eurogentec (Seraing, Belgium). The oligonucleotides containing the CPD or 6-4PP adduct were synthesized as described previously [37]. The oligonucleotides containing the C8-modified guanine derivatives—8meG, 8moG and 8brG—were synthesized as described previously [38]. The oligonucleotide containing the 1,3-d(GpNpG) cisplatin intra-strand crosslink (GNG^Pt^) was synthesized as described previously [39]. The oligonucleotides were either 5′-end-labeled with γ[^32^P]-ATP (PerkinElmer, Shelton, CT) or had the fluorescent dye Cy5 conjugated to the 5ʹ-end. To obtain duplex substrates, oligonucleotides were hybridized with corresponding complementary counterparts as described previously [40]. The resulting duplexes are referred to as N*·X, where N is a regular base in the labeled strand and X is a regular base or modified residue opposite N in the non-labeled complementary non-labeled strand. It should be stressed that the majority of data in this work were obtained with ^32^P-labeled DNA substrates, while both Cy5-labelled and radioactively labeled oligonucleotides were used to perform kinetic measurements.

**Table 1:** Sequences of the oligonucleotides used in the study.

ID ^a^	Length	Sequence (5′→3′) ^b^	Comment
*Oligodeoxyribonucleotides* ^c^			
DG1	22	CACTTCGGA**X**TGTGACTGATCC	Contains **X** at position 10, where X is a modified base [41].
cDG1-A	22	GGATCAGTCACA**A**TCCGAAGTG	Complementary to 22 mer; contains Adenine opposite damaged residue **X** [41].
RT	30	TGACTGCATA**X**GCATGTAGACGATGTGCAT	Contains **X** at position 11, where X is a modified base [42].
cRT-A	30	ATGCACATCGTCTACATGC**A**TATGCAGTCA	Complementary to RT 30 mer; contains Adenine opposite damaged residue X [42].
DG2	22	d(CACTTCGGATC**X**TGACTGATCT)	Contains **X** at position 12, where X is 8-methylGuanine (8meG) [38].
cDG2-A	22	AGATCAGTCA**A**GATCCGAAGTG	Complementary to DG 22 mer; contains Adenine at position 11 opposite **X** [38].
DG3	22	CACTTCG**X**ATCGTGACTGATCT	Contains **X** at position 8, where X is either 8-methoxyGuanine (8moG) or 8-bromoGuanine (8brG).
cDG3-A	24	AGATCAGTCACGAT**A**CGAAGTG	Complementary to DG 22 mer; contains Adenine at position 11 opposite **X**.
CPD-24	24	CTTCTTCGCAAG**XX**GGAGCTCTCT	Contains CPD adduct (XX or T = T) at positions 13-14 [34].
6-4PP-24	24	CTTCTTCGCAAG**YY**GGAGCTCTCT	Contains 6-4PP adduct (YY or T-T) at positions 13-14 [34].
c24-AA		AGAGAGCTCC**AA**CTTGCGAAGAAG	Complementary to CPD-24 and 6-4PP-24; contains AA at positions 11-12 opposite CPD and 6-4PP.
24-TG	24	CTTCTTCGCAAG**TG**GGAGCTCTCT	Complementary to c24-AA; contains TG at positions 13-14 opposite CPD and 6-4PP.
24-GT	24	CTTCTTCGCAAG**GT**GAGCTCTCT	Complementary to c24-AA; contains GT at positions 13-14 opposite CPD and 6-4PP.
24-ToG	24	CTTCTTCGCAAG**TX**GGAGCTCTCT	Complementary to c24-AA; contains **X** at position 14, where X is 8oxoG. Also contains T at positions 13 [34].
c30-AA	30	GGAGGAAGAAGTGC**AA**AGAAGAAGAGACCT	Complementary to CPD-30; contains AA at positions 15-16 opposite CPD.
30-TG	30	AGGTCTCTTCTTCT**TG**GCACTTCTTCCTCC	Complementary to c30-AA; contains TG at positions 15-16 opposite AA.
30-GT	30	AGGTCTCTTCTTCT**GT**GCACTTCTTCCTCC	Complementary to c30-AA; contains GT at positions 15-16 opposite AA.
MD2-cdA	42	AGAAACAACAGCACTACTGTACTCATG**X**ATTCTATTCCAGCA	Contains **X** at position 28, where X is 8,5′-cyclo-2′-deoxyadenosine (cdA) [43].
MD2-A	42	AGAAACAACAGCACTACTGTACTCATG**A**ATTCTATTCCAGCA	Same as MD2-cdA, but contains **A** at position 28.
MD2-G	42	AGAAACAACAGCACTACTGTACTCATG**G**ATTCTATTCCAGCA	Same as MD2-cdA, but contains **G** at position 28.
A-42	42	TGCTGGAATAGAAT**A**CATGAGTACAGTAGTGCTGTTGTTTCT	Complementary to MD2-cdA; contains A at position 15 opposite cdA.
DL-cdA	40	AATTGCTATCTAGCTCCGC**X**CGCTGGTACCCATCTCATGA	Contains **X** at position 20, where X is 8,5′-cyclo-2′-deoxyadenosine (cdA).
DL-A	40	AATTGCTATCTAGCTCCGC**A**CGCTGGTACCCATCTCATGA	Same as DL-cdA, but contains Adenine at position 20.
cDL-G	40	TCATGAGATGGGTACCAGCG**G**GCGGAGCTAGATAGCAATT	Complementary to DL-cdA and DL-A; contains mismatched G at position 21 opposite cdA and A.
cDL-A	40	TCATGAGATGGGTACCAGCG**A**GCGGAGCTAGATAGCAATT	Complementary to DL-cdA and DL-A; contains mismatched A at position 21 opposite cdA and A.
cisPt-25	25	TCTCTTCTTCT**G^**T**^G**CACTTCTTCCT	Contains 1,3-d(GpNpG) cisplatin intra-strand crosslink (GNG^Pt^) at positions 12-14 [39].
Compl-cisPt25-A	25	AGGAAGAAGTG**A**ACAGAAGAAGAGA	Complementary to cisPt-25; contains mismatched A at position 12 opposite GNG^Pt^.
Fw-MYH-G169D	30	GGTACTACTCGCGCGATCGTCGTCTGCAAG	Forward primer for site-directed mutagenesis of MUTYH.
Rv-MYH-G169D	30	CTTGCAGACGACGATCGCGCGAGTAGTACC	Reverse primer for site-directed mutagenesis of MUTYH.
Fw-MYH-G202E	32	CTTCCCGGGGTAGAGCGTTATACCGCTGGAGC	Forward primer for site-directed mutagenesis of MUTYH.
Rv-MYH-G202E	32	GCTCCAGCGGTATAACGCTCTACCCCGGGAAG	Reverse primer for site-directed mutagenesis of MUTYH.
Fw-MYH-Y165C	28	GCAGGATTAGGGTGCTACTCGCGCGGTC	Forward primer for site-directed mutagenesis of MUTYH.
Rv-MYH-Y165C	28	GACCGCGCGAGTAGCACCCTAATCCTGC	Reverse primer for site-directed mutagenesis of MUTYH.
Fw-MYH-D222N	32	GCTACTGGAGTAGTTAATGGAAATGTGGCACG	Forward primer for site-directed mutagenesis of MUTYH.
Rv-MYH-D222N	34	CACGTGCCACATTTCCATTAACTACTCCAGTAGC)	Reverse primer for site-directed mutagenesis of MUTYH.

^a^ To aid comparison with the data in the literature, the naming of oligonucleotides, when applicable, is kept consistent with previously published studies (referenced in the Comment column) in which these sequences were used. ^b^ X and Y are for modified bases, and N is for A, G, C, or T. ^c^ The relevant nucleotide context is underlined.

### 2.3. Expression and Purification of E. coli MutY and AlkA and Human MUTYH

The ORF of *MutY* and *AlkA* from *E. coli* was cloned into the pET28c(+) vector at the *Nde*I/*BamH*I sites, resulting in the expression plasmids pET28c-MutY and pET28c-AlkA, as previously described [44]. The encoded MutY and AlkA proteins carry an N-terminal 6xHis-tag sequence. The DNA sequence of the plasmid insert was verified against the sequence from the GenBank database. The expression and purification of MutY, AlkA and recombinant MUTYH proteins were performed as previously described, with minor modifications [45]. Briefly, Arctic Express (DE3) cells were transformed with the pET28b-MBP-MUTYH expression vector and plated on LB agar containing 50 µg·mL^−1^ kanamycin. A single colony was used to inoculate an overnight culture in LB supplemented with 50 µg·mL^−1^ kanamycin, which was then used to inoculate 2 L of LB medium in a flat-bottom flask. Cultures were grown at 37 °C with shaking at 180–200 rpm for 6 h until an OD_600_ of 0.6–0.8 was reached, then cooled at 4 °C for 1 h without shaking, followed by induction with 0.2 mM IPTG. Culture incubation was continued at 15 °C for 16–24 h with shaking. Cell pellets were harvested by centrifugation at 6000× *g* for 10 min at 4 °C and stored at −80 °C until cell lysis and purification.

For purification, the cell pellets were thawed and resuspended in lysis buffer (30 mM Tris-HCl, pH 7.5, 1 M NaCl, 30 mM 2-mercaptoethanol, and 5% glycerol) supplemented with a protease inhibitor cocktail (Roche Diagnostics, Mannheim, Germany). Cells were lysed using a French press at 18,000 psi, and the lysate was clarified by centrifugation at 16,000× *g* for 1 h at 4 °C. The resulting supernatant was adjusted to 250 mM NaCl and 40 mM imidazole and loaded onto a 1 mL HisTrap^TM^ Fast Flow column (Cytiva, Merck, Saint-Quentin-Fallavier, Cedex 38297, France). After sample loading, the column was washed with 20 column volumes of wash buffer (20 mM HEPES, pH 7.6, 250 mM NaCl, 40 mM imidazole, 1 mM DTT, and 5% glycerol), and the protein was eluted with elution buffer containing 500 mM imidazole. The fractions containing the protein of interest were immediately pooled and diluted tenfold with buffer A (30 mM HEPES, pH 7.6, 5% glycerol, 1 mM DTT, and 1 mM EDTA), and loaded onto a 1 mL HiTrap Heparin HP column (Cytiva, Merck). Proteins bound to the column were eluted using a linear 10–100% gradient of buffer A and buffer B, where buffer B consisted of wash buffer supplemented with 1 M NaCl. All purification procedures were carried out at 4 °C. The eluted fractions were analyzed by SDS-PAGE, and the fractions containing the pure His-tagged MBP-MUTYH, MutY and AlkA proteins were stored at −80 °C in 50% glycerol. The concentration of purified proteins was determined by the Bradford assay. In this study, we used the MBP- and His-tagged form of the MUTYH protein, without protease treatment to remove the tags. Hence, to facilitate reading of the Figures, we will refer to MBP-MUTYH simply as MUTYH.

### 2.4. DNA Glycosylase Assays

Unless indicated otherwise, DNA glycosylase activity was assayed under single-turnover conditions ([E] > [DNA]) in a buffer containing 20 mM Tris-HCl (pH 7.6), 1 mM EDTA, 100 µgml^−1^ BSA, and 30 mM NaCl at 37 °C, using 200 nM purified recombinant protein and 20 nM 5′-^32^P- or 5′-Cy-labelled oligonucleotide duplex. The reaction mixture for *E. coli* AlkA and human TDG contained 20 mM Tris–HCl (pH 8.0), 1 mM EDTA, 1 mM DTT, 100 µgml^−1^ BSA, 200 nM of protein and 20 nM DNA substrate. The reaction mixture for human uracil–DNA glycosylase (hUNGΔ84) contained 20 mM Tris–HCl (pH 8.0), 100 mM NaCl, 1 mM EDTA, 1 mM DTT, 100 µg·mL^−1^ BSA, 10 nM enzyme and 20 nM DNA substrate and was incubated for 30 min at 37 °C. All reactions were stopped by adding 10 µL of a stop solution consisting of 0.5% SDS and 20 mM EDTA. After incubation, the samples were treated with 0.1 M NaOH for 3 min at 95 °C in order to cleave AP sites left after base excision by DNA glycosylases. Then the samples were neutralized by 0.1 M HCl and desalted using a Sephadex G25 DNA grade SF column (Amersham Biosciences, 78100 Saint-Germain-en-Laye, France) equilibrated in 7.5 M urea. The desalted products of the reaction were separated by electrophoresis on denaturing 20% (*w*/*v*) polyacrylamide gels (7 M urea, 0.5 × TBE, 42 °C). The radioactive gels were exposed to a Fuji FLA-3000 Phosphor Screen, then scanned with a Typhoon FLA 9500 (Amersham Biosciences, 78100 Saint-Germain-en-Laye, France) and quantified using Image Gauge TL 1D v8.1 software. The gels containing Cy5-labelled oligonucleotides were scanned directly according to the manufacturer’s instructions. At least three independent experiments were conducted for all kinetic measurements.

### 2.5. Single Turnover Kinetics

The time courses for single-turnover (STO) kinetic experiments were performed in a large volume with 200 nM MUTYH or MutY and 20 nM substrate at 37 °C. Note that reactions were conducted under conditions assumed to be saturating based on rationale that the concentrations of MutY and MUTYH used exceeded the *K*_d_ determined for the reference substrates A·8oxoG and A·G by more than 10-fold [46,47]. At each time point, a 20 µL aliquot was withdrawn, treated and analyzed as described above. To determine the observed rate constants (*k*_obs_), the data were fitted by non-linear regression to one-phase exponential association Equation (1) using GraphPad Prism 5 software.

[Fraction product] = *A*(1 − exp(−*k*_obs_*t*)) (1)

where *A* is the amplitude, *k*_obs_ is the rate constant, and *t* is the reaction time (in minutes). Note that *k*_obs_ constants are not affected by enzyme–substrate association or by product inhibition, so that *k*_obs_ reflects the maximal base excision rate (*k*_obs_ ≈ *k*_max_). Here, we assume that the conditions used correspond to [E] >> [S] > *K*_d_. However, because *K*_d_ values were not determined for all substrates, *k*_obs_ values at the tested enzyme concentration are reported as apparent rate constants and used for relative comparison of substrate processing efficiency.

### 2.6. Structural Modeling and Conservation Analysis

AlphaFold3 [48] was used to generate models of full-length human MUTYH and *E. coli* MutY, either free or bound to various mismatch-containing DNA based on the sequence from the 8FAY structure [49]. Additionally, *E. coli* MutY bound to DNA from the 3G0Q structure was modeled to ensure that the predictions are independent of the DNA sequence used. Five top-scoring models were considered for each simulation. For MUTYH, full-length models of the free protein were generated first, their low-confidence N- and C-terminal tails were mapped and discarded, and the DNA-bound models were regenerated for the high-confidence core (residues 56–476), encompassing both the catalytic domain and the NUDIX domain (Supplementary Figure S1; Supplementary File S1).

MUTYH/MutY docking to damaged DNA was performed with pyDockDNA [50] in a no-desolvation mode using coordinates of thymine dimer-containing (1TTD, 1VAS [51,52]) and GTG^Pt^ DNA (1DA4, 1DA5 [53]) as starting structures. As controls, structures of GsMutY from its complexes with pre-eversion and post-eversion A·8oxoG mispairs (5KN9 and 3G0Q, respectively [7,54]) were docked back onto the DNA part of the same complex. The orientation of the protein on various DNAs was estimated using the following distances as guides: for the post-eversion complexes, from N[Thr205/Thr121/Thr127] (MUTYH/*E. coli* MutY/GsMutY) to O1P of the 2nd phosphate 3′ of the target A (2.85 Å in 3G0Q) and from N[Thr474/Ala335/Val346] to O1P of the 1st phosphate 3′ of the opposite nucleotide (2.82 Å in 3G0Q); for the pre-eversion complexes, from N[Thr205/Thr121/Thr127] to O1P of the 2nd phosphate 3′ of the target A (2.96 Å in 5KN9) and from N[Tyr165/Tyr82/Tyr88] to O1P or O2P (whichever is less) of the 2nd phosphate 3′ of the opposite nucleotide (2.97 Å in 5KN9).

Conservation of key residues in MutY homologs was visualized in WebLogo v3.9 [55] using a taxonomically balanced set of sequences with the representation of one sequence per class (Supplementary File S2). The sequences were retrieved from the NCBI RefSeq database by BLASTp search [56] with human MUTYH, *Arabidopsis thaliana* MYH, *Schizosaccharomyces pombe* Myh1 and *E. coli* MutY as queries, which were filtered to discard those lacking the iron–sulfur cluster and a C-terminal extension, and aligned using Clustal Omega [57].

## 3. Results

### 3.1. The Purified MUTYH and MutY Proteins Exhibit Classical DNA Glycosylase Activities

**Figure 1:**
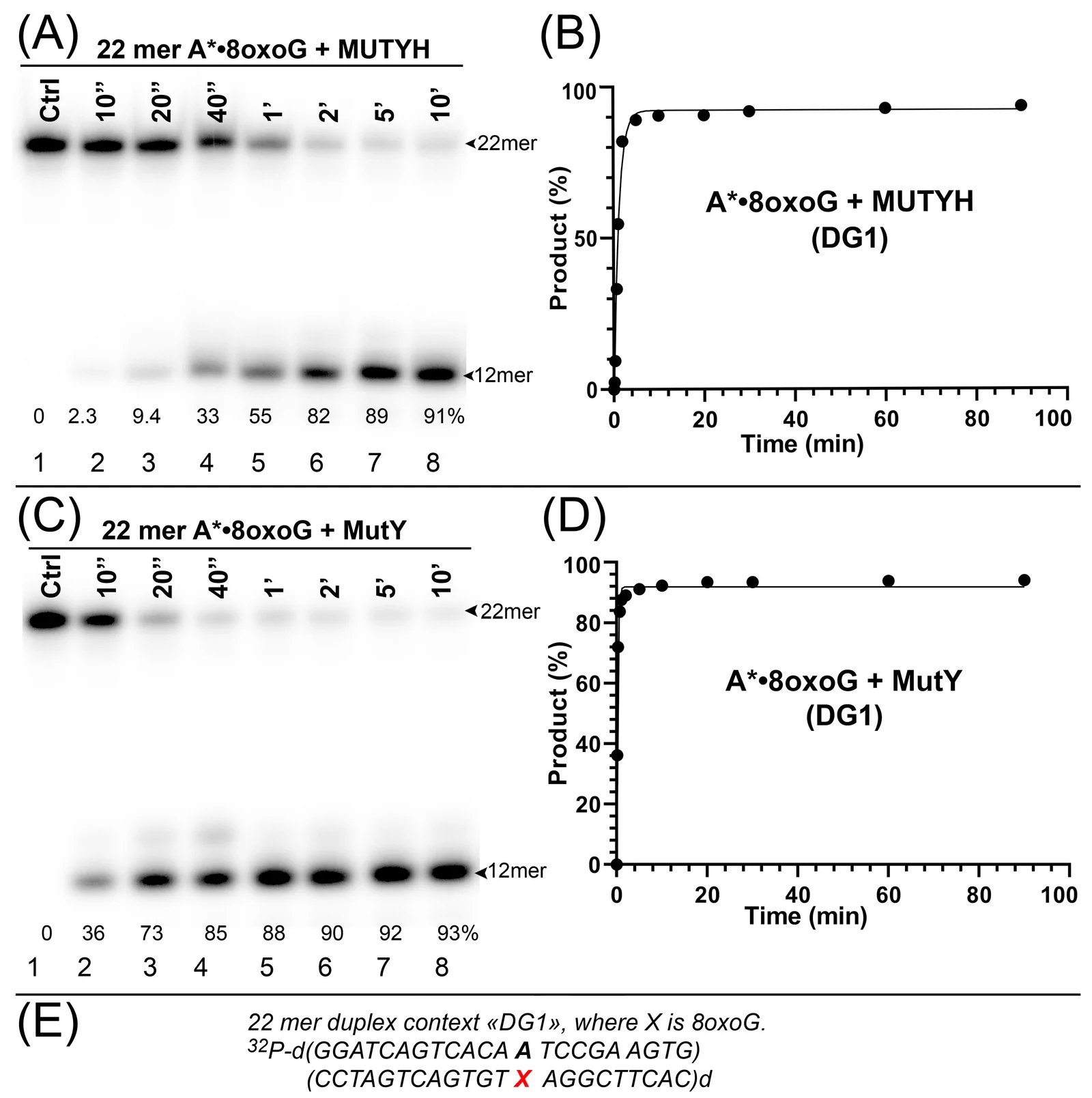
Time-dependent adenine excision in A·8oxoG oligonucleotde duplex (context «DG3») by MUTYH (**A**,**B**) and *E. coli* MutY (**C**,**D**). In total, 20 nM of the 22 mer 5′-^32^P-labeled A*·8oxoG duplex oligonucleotide was incubated under single turnover conditions with 200 nM MUTYH or MutY proteins for varying periods of time at 37 °C. Arrows mark the size of the DNA substrate and the cleavage fragments. Quantities above the lane numbers (lanes 1–8) correspond to the percentage of cleavage products. (**B**,**D**) Plots of pre-steady-state single turnover kinetics of MUTYH- and MutY-catalyzed cleavage of 22-mer A·8oxoG duplexes. Mean ± SD of three independent experiments is shown. (**E**) Schematic representation of the 22-mer duplex sequence with the target adenine residue and opposite modified base. For details, see Section 2.

While the basic catalytic machinery and motifs recognizing A and 8oxoG are conserved among MutY homologs across all domains of life (Supplementary Figure S2A–D), human MUTYH is sufficiently remote from well-characterized bacterial homologs (Supplementary Figure S2G) to be expected to possess unique features, especially concerning non-canonical activities. Using previously established methods [44,49], we successfully expressed and purified recombinant His-tagged MBP-MUTYH and MutY proteins, which were then used to examine DNA glycosylase activities on a canonical DNA substrate containing an A·8oxoG base pair. Note that for ease of reading, we will refer to MBP-MUTYH simply as MUTYH throughout the article. Here, we used 20 nM of an A*·8oxoG DNA duplex (22 mer), where the A-containing strand was ^32^P-labeled. The reaction was carried out with a 10-fold molar excess of MUTYH or MutY (200 nM) for varied periods at 37 °C. As shown in Figure 1, MutY and MUTYH exhibit highly efficient excision of adenine in the 22 mer A*·8oxoG duplex and generate a 14 mer cleavage product after hot alkaline treatment, indicating that DNA glycosylases generate an abasic site after the excision of the target base. MUTYH was able to excise ~80% of adenine in 2 min (Figure 1A, lane 6), whereas MutY reached the same extent of adenine excision after only 20 s (Figure 1C, lane 3), suggesting that the bacterial enzyme is more efficient compared to its human counterpart under the experimental conditions tested, as reported [49,58].

**Figure 2:**
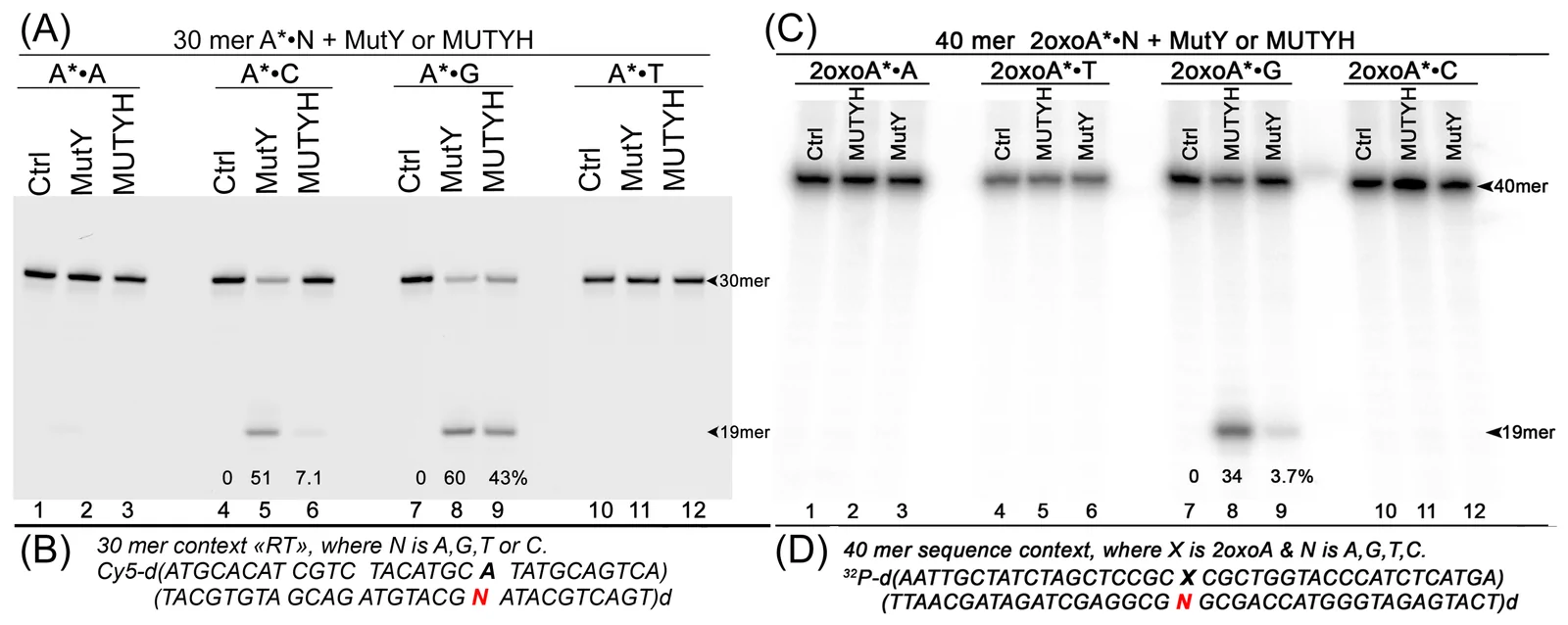
Activities of MUTYH and MutY proteins towards duplex oligonucleotides containing either (**A**) adenine or (**C**) 2-hydroxyadenine residue (2oxoA) opposite four regular nucleotides. In total, 20 nM of 30-mer 5′-Cy5-labelled A*·A, A*·C, A*·G and A*·T or 40-mer 5′-[^32^P]-labeled 2oxoA*·A, 2oxoA*·T, 2oxoA*·G and 2oxoA*·C oligonucleotide duplexes were incubated with 200 nM of MUTYH and MutY for 1 h at 37 °C. Arrows indicate the sizes of the DNA substrate and cleavage fragments. The quantities designated above the lane numbers (panel A: lanes 4 to 9 and panel C: lanes 7 to 9) correspond to the percentage of cleavage products. (**B**,**D**) Schematic representations of 30-mer (context “RT”) and 40-mer (context “DL”) duplex sequences with the target residue in bold and the opposite regular base in red. For details, see Section 2.

Next, we examined MUTYH and MutY activities towards adenine residues opposite four regular nucleobases. For this, 20 nM of 30-mer 5′-Cy5-labelled A·A, A·C, A·G and A·T oligonucleotide duplexes were incubated with 200 nM MutY or MUTYH for 1 h at 37 °C. As shown in Figure 2A, in agreement with previous observations, we observed efficient cleavage of the A·G duplex and generation of the expected 19-mer cleavage product by both human and bacterial DNA glycosylases, indicating the excision of mismatched adenine at position 20 [59,60]. Bacterial MutY also exhibited robust activity towards the A·C duplex, whereas human MUTYH excised A opposite C with very low efficiency. These results confirm that human DNA glycosylase exhibits lower activity towards regular base mismatches compared to bacterial MutY. Thus, both purified enzymes were highly active on their classical DNA substrates and hence can be used to check their activities towards other types of DNA damage. Interestingly, the bacterial enzyme preparation showed relatively higher activity toward classical DNA substrates than its human counterpart, possibly due to the greater stability of the MutY protein during prolonged incubation (up to 18 h at 37 °C) compared with MUTYH under the experimental conditions used.

To further characterize our enzyme preparations using established DNA substrates, we examined DNA glycosylase activities of MUTYH and MutY towards oligonucleotide duplexes containing the oxidized adenine residue 1,2-dihydro-2-oxoadenine (2oxoA) opposite four regular nucleobases. In agreement with previous observations [61], human DNA glycosylase excised 34% of DNA containing 2oxoA opposite guanine (Figure 2C). In contrast, the bacterial enzyme exhibited only 3.7% product conversion. We did not detect any activity of MUTYH and MutY on 2oxoA·A, 2oxoA·T and 2oxoA·C duplexes.

These results further substantiate the particular DNA substrate specificity of human DNA glycosylase towards the 2oxoA residue in duplex DNA, suggesting that misincorporation of the 2oxodATP precursor opposite G during DNA replication presents a significant threat to genome integrity in mammalian cells and possibly in other eukaryotes.

### 3.2. Action of MUTYH and MutY Adenine-DNA Glycosylases Towards Oligonucleotide Duplexes Containing Mismatched Adenine Residue Opposite Non-Bulky Base Modifications

**Figure 3:**
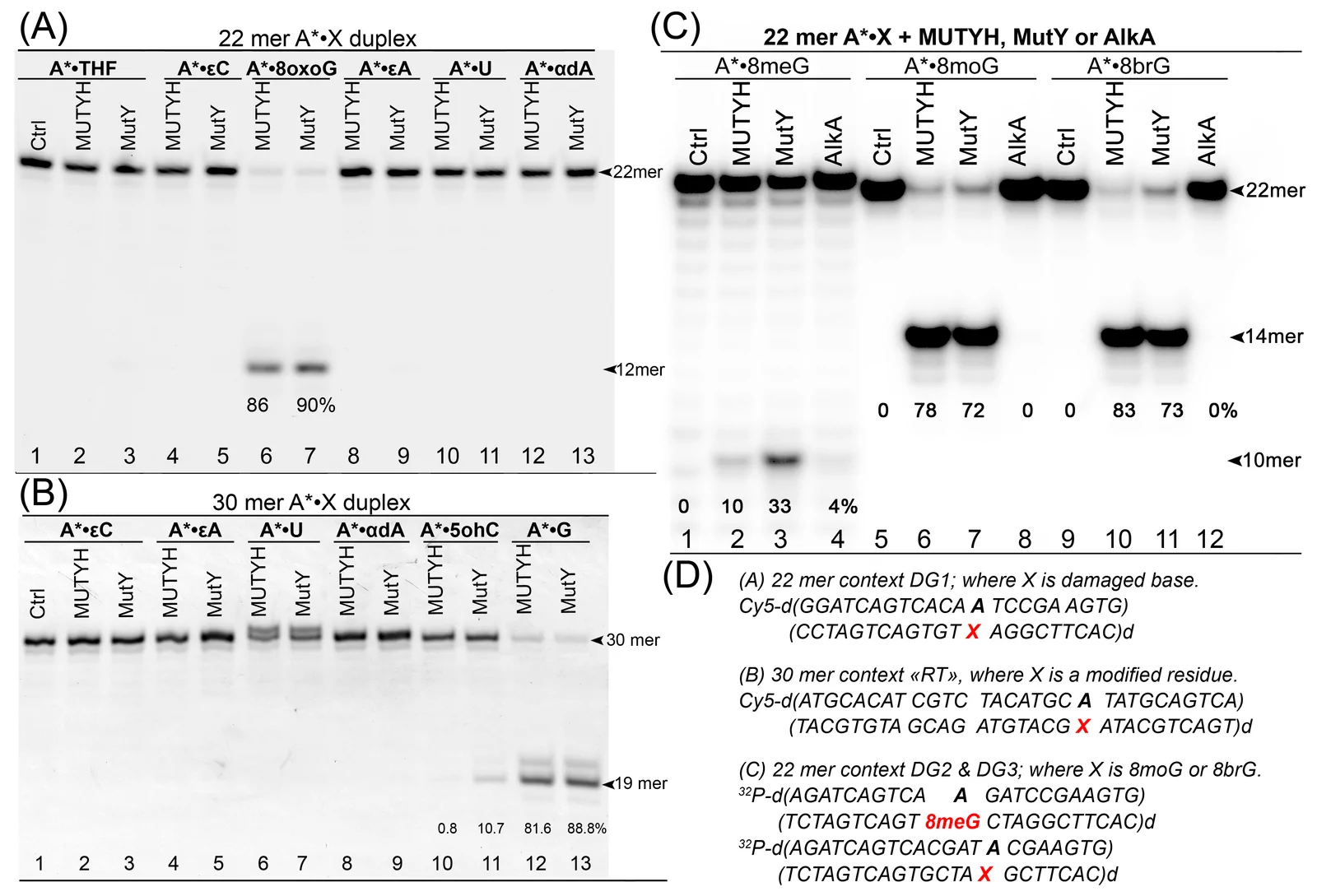
Activities of MUTYH, MutY and AlkA proteins towards duplex oligonucleotides containing mismatched adenine opposite various base modifications. (**A**,**B**) A*·X oligonucleotide duplexes (context “22” and “RT”) containing non-bulky base modifications. In total, 20 nM of short 22-mer or 30-mer 5′-Cy5-labeled A*·THF, A*·εC, A*·8oxoG, A*·εA, A*·U and A*·αdA oligonucleotide duplexes were incubated with 200 nM of MutY or MUTYH for 1 h at 37 °C. (**C**) Activities of MUTYH, MutY and AlkA proteins towards duplex oligonucleotides containing mismatched adenine opposite C8-modified guanine derivatives. Briefly, 20 nM of short 22-mer 5′-^32^P-labeled A*·8meG, A*·8moG and A*·8brG oligonucleotide duplexes were incubated with 200 nM of MUTYH, MutY and AlkA for 1 h at 37 °C. Arrows indicate the sizes of the DNA substrate and cleavage fragments. The quantities designated above the lane numbers (Panel A: lanes 6–7; Panel B: lanes 10–13; and Panel C: lanes 1–12) correspond to the percentage of cleavage products. For details, see Section 2. (**D**) Schematic representation of sequences of 22-mer and 30-mer duplexes with the target adenine residue in bold and opposite modified DNA bases in red.

The novel activity of MutY and MUTYH on A opposite 8moG and 8meG identified herein suggests that these DNA glycosylases can process other types of substrates. To further substantiate our understanding of MutY/MUTYH substrate preference, we investigated the aberrant excision of A opposite various subtle base modifications such as apurinic/apyrimidinic (AP) site analog, tetrahydrofuran (THF), 3,*N*^4^-ethenocytosine (εC), 1,*N*^6^-ethenoadenine (εA), uracil (U), 5-hydroxycytosine (5ohC) and alpha-anomeric 2′-deoxyadenosine (αdA). Note that we placed mismatched adenine in short 22-mer or 30-mer oligonucleotide duplexes with different sequence contexts. As shown in Figure 3A and as expected, incubation of the 22-mer A·8oxoG duplex with MUTYH and MutY generated 12-mer cleavage products, indicating the excision of A opposite 8oxoG (lanes 6–7). However, there were no measurable cleavage products with other DNA substrates containing A opposite THF, εC, εA, U and αdA by human and bacterial adenine-DNA glycosylases (lanes 2–5 and 8–13). This suggests that aberrant MutY/MUTYH activities are restricted to A opposite modified Gs.

Next, we searched for the presence of aberrant activities using 30-mer duplexes with different sequence contexts. For this, we incubated 20 nM of 30-mer 5′-Cy5-labeled A*·εC, A*·εA, A*·U, A*·αdA, A*·5ohC and A*·G oligonucleotide duplexes in the presence of 200 nM MutY and MUTYH for 1 h at 37 °C. After the reactions, we analyzed the products by denaturing PAGE. As shown in Figure 3B and as expected, incubation of the 30-mer A*·G duplex with MUTYH and MutY generated 19-mer cleavage products, indicating the excision of adenine opposite G (lanes 12–13). Once again, we did not observe cleavage products with other 30-mer DNA substrates, indicating that MUTYH and MutY do not excise adenine residues opposite ethenobases, uracil and alpha-anomeric nucleotides (lanes 2–9). Nevertheless, incubation of the 30-mer A*·5ohC duplex with MutY generated a weak 19-mer cleavage fragment (lane 11), suggesting excision of A opposite oxidized cytosine, 5ohC, although this activity is much lower compared to the A*·G duplex (lane 13). In contrast, MUTYH exhibited very weak or no activity towards the A*·5ohC duplex (lane 10).

Taken together, these results suggest that human and bacterial adenine-DNA glycosylases lack aberrant activities towards DNA duplexes containing various base modifications other than that of 8oxoG. Nevertheless, based on our data we may suggest that bacterial MutY exhibits less restraint and wider DNA substrate specificities compared to its human counterpart.

### 3.3. Human and Bacterial Adenine-DNA Glycosylases Can Excise Adenine Opposite C8-Modified Guanine Derivatives

C8-modified guanine derivatives such as 8-methylguanine (8meG) and 8-methoxyguanine (8moG) residues can be generated by DNA alkylation with carbon-centered free radicals. 8meG is weakly mutagenic, but can induce G→C transversions, being associated with the carcinogenic effect of 1,2-demethylhydrazine in colon cancer in rodents [62]. C8-halogenated guanine such as 8-bromoguanine (8brG) can be generated by reactive brominating species produced during inflammation, where bromide-derived oxidants can attack guanine at the C8 position [63]. 8brG is mutagenic because it can alter base-pairing during replication and induce mainly G→T mutations in human cells [64]. Previously, it was shown that 8meG is excised by *E. coli* 3-methyladenine-DNA glycosylase II (AlkA) [38], whereas no DNA glycosylase has been identified for the excision of 8brG and 8moG in bacterial and human cells [64]. Nevertheless, it was found that MUTYH and MutY can excise A from A·8brG mismatches and opposite other C8-modified guanine derivatives, thus preventing mutations in the newly synthesized strand during DNA replication [45,64,65].

Both bacterial and mammalian adenine-DNA glycosylases are highly specialized in the repair of A·8oxoG, but it was unknown whether these enzymes could excise adenine opposite C8-modified guanine derivatives other than 8oxoG, such as 8meG and 8moG. Here, we examined whether MutY, MUTYH and AlkA can excise A opposite C8-modified guanine derivatives. For this, the reactions were carried out under single-turnover conditions as mentioned above. Notably, AlkA was used here to study its possible role as a back-up enzyme for MutY, since it was previously shown that AlkA can excise regular purines from mismatches [66]. As shown in Figure 3C, incubation of MUTYH and MutY with A·8moG- and A·8brG-containing DNA duplexes, followed by hot alkaline treatment, resulted in measurable cleavage products indicating the formation of an AP site after the excision of A. Thus, both MutY and MUTYH excised adenine opposite 8moG and, as expected, opposite 8brG with efficiencies of >40% and 8meG with 10 and 33% for MUTYH and MutY, respectively. Notably, incubation of bacterial AlkA with A·8moG and A·8brG duplexes did not show product cleavage (lanes 8 and 12). Nevertheless, we observed weak activity on A*·8meG with 4% cleavage. Previously, it was shown that AlkA can excise G from the G*·A mismatch, but with compromised recognition of adenine opposite G in the A*·G mismatch [66]. Therefore, our results confirm these observations that AlkA does not or very weakly recognizes A opposite C8-modified guanine under the experimental conditions used.

To quantitatively assess the efficiency of bacterial and human adenine-DNA glycosylases, we measured the time-dependent kinetics of MUTYH and MutY-mediated cleavage of 22-mer (“DG” sequence context) oligonucleotide duplexes A·8oxoG, A·G, A·8meG, A·8moG, and A·8brG under single-turnover conditions. Note that in this experiment, we used the “DG” sequence context (Table 2 and Supplementary Figures S3–S7). Based on these kinetic analyses, we determined *k*_obs_ values of MutY and MUTYH-catalyzed adenine excision activities and then compared them to *k*_obs_ of excision of A in an A·8oxoG duplex with the same sequence context and adenine positions as in the A·G, A·8moG, and A·8brG duplexes. As shown in Table 2, when using A*·G, A*·8meG, A*·8moG, and A*·8brG DNA duplexes, the *k*_obs_ values of MUTYH-catalyzed excision of A were 8, n.d., 4.4 and 8-fold lower, respectively, than that of A in the A*·8oxoG duplex (0.79 min^−1^). For MutY, the *k*_obs_ values were 31, 430, 48, and 36-fold lower compared to the canonical substrate, respectively. These results suggest that both human and bacterial adenine-DNA glycosylases exhibit a strong preference for the A*·8oxoG substrate. Nevertheless, MutY and MUTYH were able to repair adenine opposite C8-modified guanine derivatives, except 8meG, with relatively good efficiency comparable to that of the A*·G duplex, suggesting a potential biological role in the prevention of mutations induced by unrepaired 8moG and 8brG residues in DNA.

### 3.4. Human MUTYH and E. coli MutY Do Not Remove Adenine Opposite Bulky UV Adducts in Duplex DNA

**Figure 4:**
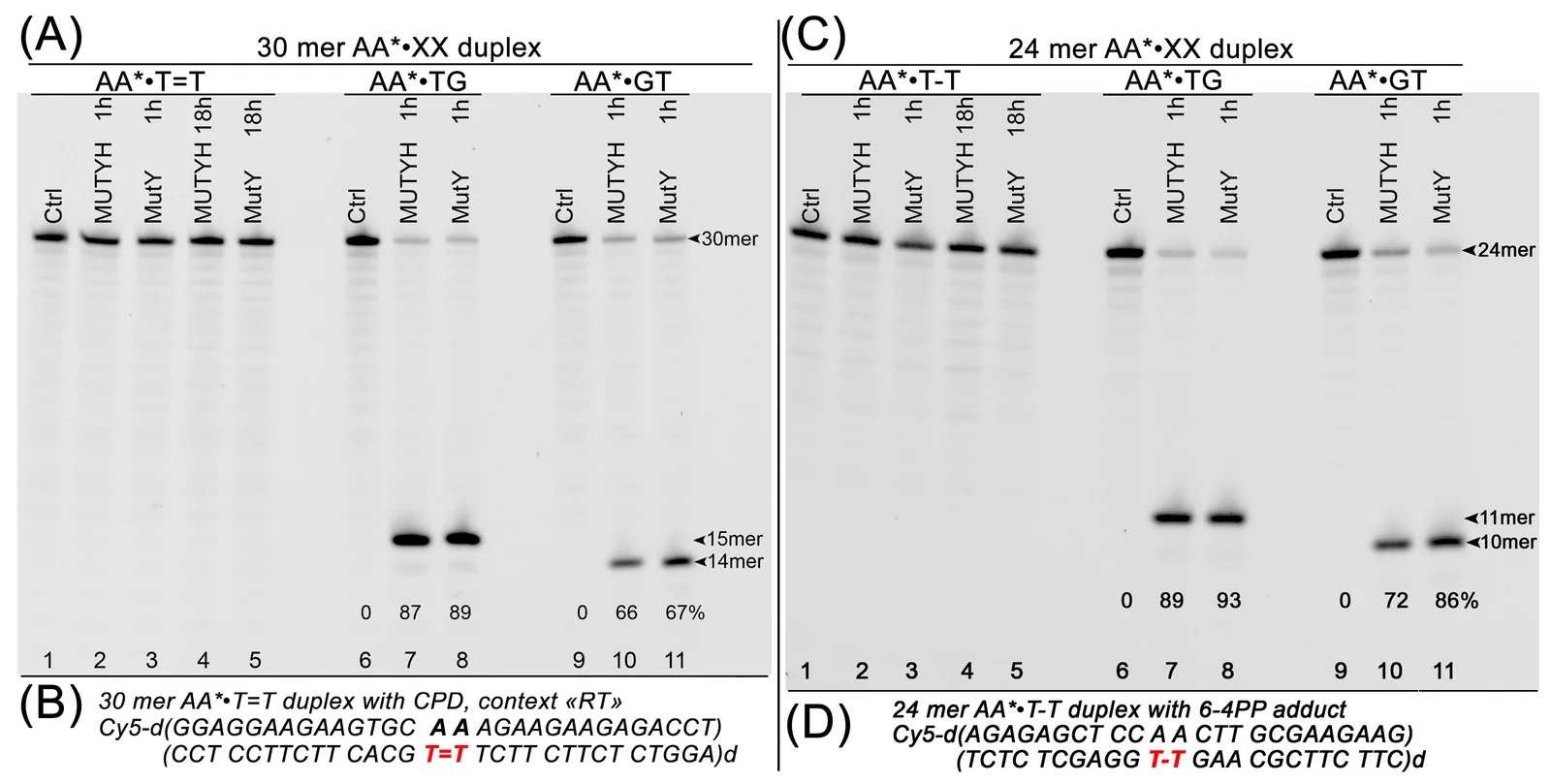
Action of MUTYH and MutY on oligonucleotide duplexes containing UV pyrimidine dimers. In total, 20 nM of 5′-Cy5-labeled (**A**,**B**) 30-mer A*·G, AA*·T = T and (**C**,**D**) 24-mer AA*·T-T, A*·G duplexes were incubated in the presence of 200 nM MUTYH and MutY for 1 h or 18 h at 37 °C. (**A**,**C**) Arrows indicate the sizes of the DNA substrate and cleavage fragments. The quantities designated above the lane numbers (lanes 6–11 in panels A and C) correspond to the percentage of cleavage products. For details, see Section 2. (**B**,**D**) Schematic representation of 30-mer and 24-mer duplex sequences with the target adenine residue in bold and the opposite UV pyrimidine dimer in red.

Previously, we showed that bacterial MutY was not able to excise adenine opposite UV-induced cyclobutane pyrimidine dimers (CPD, hereinafter referred to as T = T) and 6-4PP (hereinafter referred to as T-T) [34]. To investigate whether human MUTYH might initiate aberrant repair when acting upon UV-induced DNA damage, 20 nM of 5′-Cy5-labeled 30-mer AA*·T = T and 24-mer AA*·T-T duplexes (in which the non-damaged DNA strand is labeled at 5′ with Cy5 fluorescent dye and contains A* opposite the adduct) (see Figure 4B,C) were incubated in the presence of a molar excess of human MUTYH and bacterial MutY proteins (200 nM) for 1 h and 18 h at 37 °C.

To define the position of the site of cleavage in the regular strand, we produced DNA size markers from 30-mer and 24-mer duplexes labeled with a 5′-Cy5 group. These duplexes contained a single A·G mismatch located at either position 15 or 16 (for the 30-mer duplex) or position 11 or 12 (for the 24-mer duplex), designated hereinafter as AA*·TG or AA*·GT. After incubation with DNA glycosylases, the end-product AP sites were incised using light piperidine treatment and the reaction products were analyzed on a denaturing PAGE gel. As expected, incubation of 30-mer and 24-mer AA·TG or AA·GT duplexes with MUTYH and MutY generated either 15- and 14-mer cleavage fragments (Figure 4A) or 11- and 10-mer cleavage fragments (Figure 4C), respectively. These results indicate that MUTYH and MutY excise mismatched A residues opposite G, as expected. Notably, no products of cleavage were detected after incubation of 30-mer AA·T = T and 24-mer AA·T-T duplexes for 1 h and 18 h at 37 °C with the bacterial and human adenine-DNA glycosylases. These results indicate that MUTYH and MutY are both incapable of excising A residues opposite 6-4PP and CPD lesions in both 30-mer and 24-mer duplexes.

### 3.5. Action of MUTYH and MutY Towards Oligonucleotide Duplexes Containing Adenine Opposite Helix-Distorting DNA Adducts

8,5′-*cyclo*-2′-deoxypurines such as diastereoisomeric (5′*S*)- and (5′*R*)-8,5′-*cyclo*-2′-deoxyadenosine (cdA) are produced in the absence of oxygen through the attack of a hydroxyl radical on the C5′ carbon of the sugar via hydrogen atom abstraction. This results in the formation of a radical centered on the sugar’s C5′ carbon, which subsequently reacts with the purine at the C8 carbon. The presence of the cdA adduct in a DNA duplex causes significant alterations to the backbone torsion angles, leading to a weakening of hydrogen bonds between base pairs and severe disruptions to the helical conformation in the vicinity of the adduct. Notably, *S*-cdA diastereoisomers accumulate in non-treated mouse organs, possibly due to inefficient repair [67,68]. On the other hand, cisplatin, a widely used anticancer drug, interacts with purine residues at the nucleophilic reactive centers N7 or O6 to form DNA crosslinks. The vast majority of formed DNA adducts are 1,2-intrastrand crosslinks (65% at 5′-GG and 25% at 5′-AG), and 5% are 1,3-intrastrand crosslinks at d(GpNpG) [69,70]. In general, a DNA duplex with a 1,3-intrastrand crosslink at 5′-GNG is a locally kinked and unwound, with major perturbation near the platinum-linked guanines.

**Figure 5:**
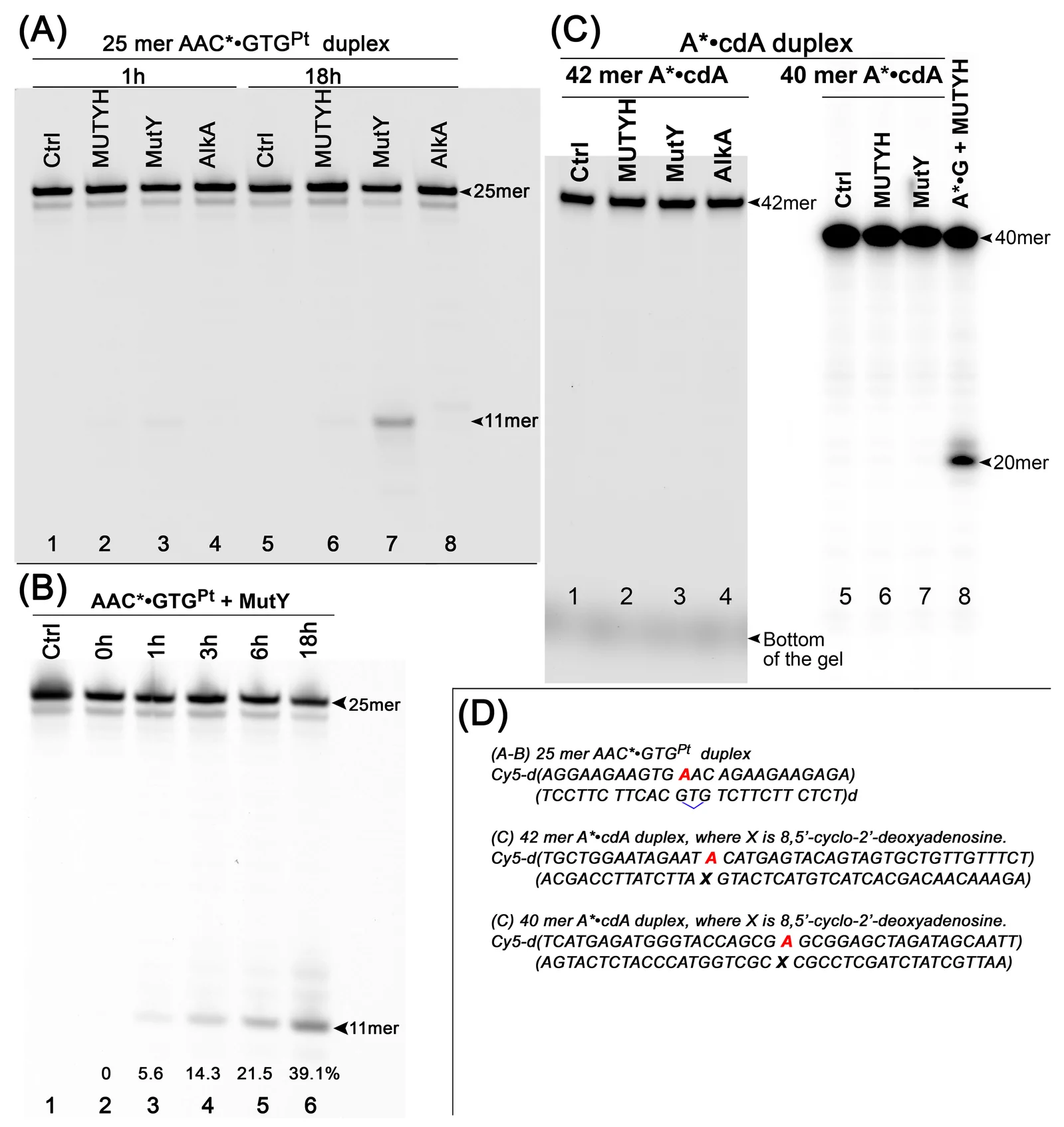
Action of MUTYH, MutY and AlkA on oligonucleotide duplexes containing adenine opposite either 8,5′-*cyclo*-2′-deoxyadenosine (cdA), regular guanine or 1,3-d(GpNpG) cisplatin intra-strand crosslink (GTG^Pt^). (**A**,**B**) In total, 20 nM of 5′-Cy5-labeled 25 mer AAC*·GTG^Pt^ oligonucleotide duplex or (**C**) 20 nM of either 42-mer 5′-Cy5-labeled A*·cdA or 40-mer 5′-^32^P-labeled A*·cdA and A*·G oligonucleotide duplexes were incubated in the presence of 200 nM MUTYH, MutY and AlkA for 1 h at 37 °C. Arrows indicate the sizes of the DNA substrate and cleavage fragments. The quantities designated above the lane numbers (lanes 2–6 in Panel B) correspond to the percentage of cleavage products. For details, see Section 2. (**D**) Schematic representation of 25-mer, 42-mer and 40-mer duplex sequences with the target adenine residue in red and opposite modified residues or guanine in bold.

Here, we studied the action of MUTYH, MutY and AlkA on a DNA duplex containing mismatched adenine opposite a 1,3-d(GpTpG) cisplatin intra-strand crosslink (GTG^Pt^). In total, 20 nM of 5′-Cy5-labeled 25-mer AAC*·GTG^Pt^ oligonucleotide duplex was incubated in the presence of 200 nM MUTYH, MutY or AlkA for varying periods of time at 37 °C. As shown in Figure 5A, no cleavage product was observed after 1 h of incubation; however, after a long 18 h incubation of the AAC*·GTG^Pt^ duplex with MutY, we observed the formation of a cleavage fragment (lane 7), indicating excision of the adenine residue opposite 3′-guanine in GTG^Pt^. No cleavage of the AAC*·GTG^Pt^ duplex was observed after 1 h and 18 h incubations with MUTYH and AlkA. Next, we examined the time-dependent excision of adenine opposite GTG^Pt^ by MutY. As shown in Figure 5B, after 1, 3, 6 and 18 h of incubation at 37 °C, we observed a linear increase in the generation of the 11-mer cleavage fragment after 1, 3, 6 and 18 h of incubation at 37 °C in the presence of MutY. Based on these results, we estimated *k*_obs_ values of MutY-catalyzed excision of adenine opposite the cisplatin adduct (0.04 ± 0.02 min^−1^), which is 108-fold lower than *k*_obs_ of the excision of A in an A*·8oxoG duplex (Table 2). This unexpected result suggests that bacterial adenine-DNA glycosylase upon long incubation time is prone to aberrant excision of adenine residues under certain sequence contexts and types of DNA damage, contrary to its human counterpart.

Next, we examined the action of MUTYH, MutY and AlkA on a DNA duplex containing adenine opposite cdA adduct. As shown in Figure 5C, no cleavage products were observed after incubation of A•cdA duplexes with 200 nM MUTYH and MutY for 1 h at 37 °C, indicating that the DNA glycosylases do not recognize adenine opposite the bulky cdA adduct.

### 3.6. Purification and Characterization of Mutant Variants of the Human MUTYH Protein

The main objective of our study was to examine the activities of MUTYH and MutY using a non-canonical DNA substrate in order to identify a putative aberrant excision of adenine residues in duplex DNA when opposite base modifications differ from that of 8oxoG. It should be stressed that previously, several laboratories demonstrated that specific amino acid changes in the active sites of DNA glycosylases could result in a dramatic increase in the aberrant excision of regular bases in non-damaged DNA duplexes by the mutant enzymes. For example, mutant human uracil-DNA glycosylases Y145A and Q204D can excise thymine and cytosine in T·A and C·G base pairs, respectively [71]. Meanwhile, human TDG-A145G and -H151A mutants exhibit substantial aberrant activities towards the T·A base pair in regular DNA duplexes [72]. Given that alterations in substrate recognition by DNA glycosylases can result from mutations affecting the active site or the lesion-recognition surface, we sought to determine whether cancer-associated MUTYH variants exhibited altered specificity toward non-canonical DNA substrates. To this end, we investigated the DNA glycosylase activities of mutant MUTYH variants using both standard A·8oxoG DNA substrates and non-canonical substrates.

**Figure 6:**
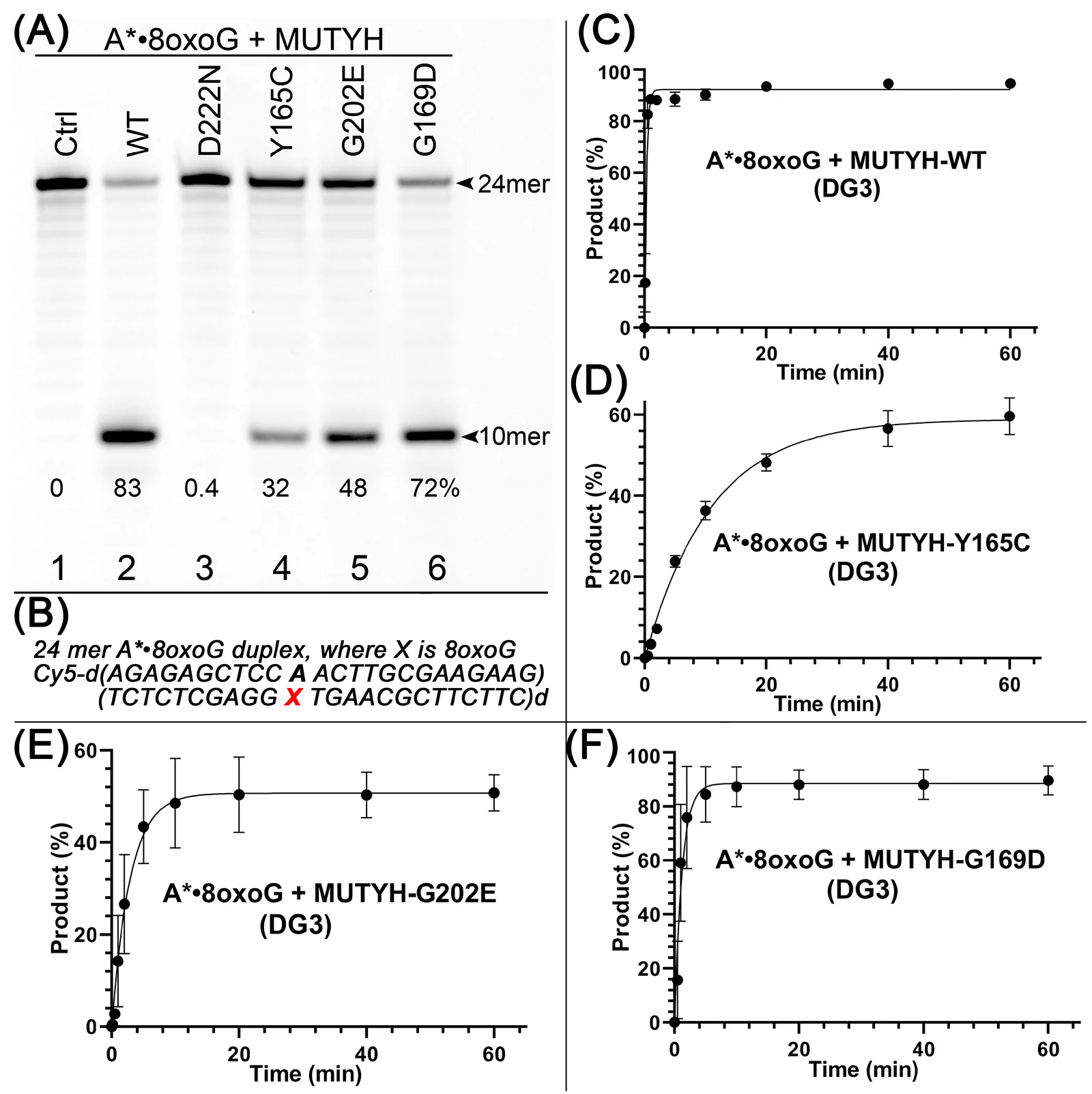
Action of MUTYH wild-type and mutant variants towards A*·8oxoG duplex. (**A**) Denaturing PAGE analysis of reaction products. Time courses were performed using 20 nM of 22-mer 5′-Cy5-labeled A*·8oxoG duplex oligonucleotide and 200 nM of MUTYH WT, D222N, Y165C, G202E and G169D proteins for 1 h at 37 °C. Arrows indicate the sizes of the DNA substrate and cleavage fragments. The quantities designated above the lane numbers (Panel A: lanes 1–6) correspond to the percentage of cleavage products. For details, see Section 2. (**B**) Schematic representation of the 24-mer duplex sequence (context «DG1») with the target adenine residue in bold and the opposite 8oxoG residue in red. (**C**–**F**) Plots of the pre-steady-state single turnover kinetics of MUTYH variant-catalyzed cleavage of the A*·8oxoG duplex oligonucleotide. Mean ± SD of three independent experiments is shown. For details, see Section 2.

Of more than 1000 MUTYH missense variants identified in the human population, the best-studied are Y165C and G382D, two founder mutations conferring susceptibility to polyposis and colorectal cancer in the European population. Tyr165 is highly conserved among MutY homologs and has a well-established mechanistic role as a wedge inserted into DNA to kink it, while Gly382, also participating in the kinking structure, is somewhat less conserved (Supplementary Figure S2E,F). However, the majority of the MUTYH missense mutations are variants of uncertain significance (VUSs), whose effects on enzyme activity and disease risk are not clearly understood. Note that four different MUTYH residue numerations can be found in the literature; here, we stick to the original one from [73] (Supplementary Figure S8). Having established a well-working expression technology for the human adenine-DNA glycosylase, we have purified four *MUTYH* variants, including D222N (catalytically inactive variant), Y165C, G169D and G202E, for further biochemical characterization (Supplementary Figure S9). The D222N and Y165C variants have already been characterized and were used as catalysis-negative controls in this study. The other two variants, MUTYH-G169D and MUTYH-G202E, are VUSs. The DNA glycosylase activities of purified MUTYH variants were measured using the classical substrate A*·8oxoG and non-canonical DNA substrates such as A*·C, A*·5ohC, A*·THF and AA*·T = T duplexes (Supplementary Figures S10 and S11). To qualitatively evaluate the classical activity of MUTYH variants, 20 nM of 5′-Cy5-labeled 22-mer A*·8oxoG duplex oligonucleotide was incubated in the presence of a molar excess of wild-type and mutant variants of MUTYH (200 nM) for 1 h at 37 °C. As shown in Figure 6A, incubation of the A*·8oxoG duplex with most proteins, except the D222N mutant (lane 3), generated the expected 12-mer cleavage fragment with varying efficiencies (lanes 2, 4–6).

Next, using the same 22-mer A*·8oxoG duplex oligonucleotide, we performed more sensitive time-dependent kinetics to compare, in a quantitative manner, the enzymatic activities of mutants to that of wild-type (Figure 6C–E and Supplementary Figure S10). As shown in Table 3, all variants exhibited reduced adenine-DNA glycosylase activity compared to MUTYH-WT in the following order: WT > G169D > G202E >> Y165C >> D222N. Among the VUSs tested, G169D retained substantial residual activity (~26% of WT activity), whereas G202E displayed a more pronounced catalytic defect and retained only ~11% of WT activity.

Finally, we tested the purified MUTYH variants against A*·G, A*·C, A*·5ohC, A*·THF and AA*·T = T duplexes to determine whether cancer-associated mutations may stimulate aberrant activities. The results revealed that MUTYH mutants do not exhibit aberrant activities towards the non-canonical DNA substrates tested, suggesting that the cancer risk is due to a decrease in the classical activity of MUTYH towards A*·8oxoG mispair in cellular DNA (Supplementary Figure S11).

### 3.7. Structural Modeling of MUTYH

Intrigued by the phenomenon of aberrant repair activity among mismatch-specific DNA glycosylases upon interaction with damaged DNA duplexes, we explored possible structural reasons underlying this phenomenon. Among MutY homologs, *Geobacillus stearothermophilus* MutY (GsMutY) is the most extensively structurally characterized, providing a series of enzyme–DNA complexes from the initial recognition of an A·8oxoG pair through a series of reaction intermediates to the complex with the AP site reaction product [49,54,74,75]. For human MUTYH, only the structures of the free catalytic domain and a full-length protein bound to DNA containing a transition state analog have been solved [49,76], whereas *E. coli* MutY and several of its mutants have been characterized only as free proteins or in a complex with the adenine base [54,77]. Hence, we took advantage of the ability of AlphaFold 3 to build models of protein–DNA complexes, including those with some chemical modifications such as 8-oxoG, to assess possible structures of MUTYH and MutY bound to the substrates on which the aberrant activity was found.

**Figure 7:**
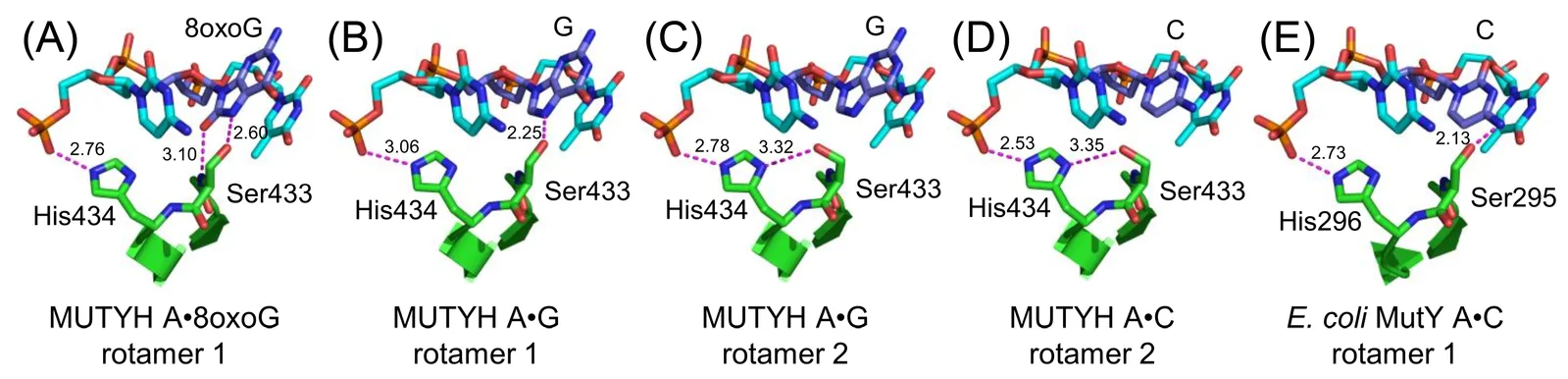
Rotamers of the Ser residue recognizing the base opposite the target A in the AlphaFold 3 models generated for the protein–DNA complexes. (**A**–**D**) MUTYH bound to DNA containing A·8oxoG (**A**), A·G (**B**,**C**) and A·C (**D**) mispairs; *E. coli* MutY bound to DNA containing an A·C mispair (**E**). Interatomic distances in Å are indicated.

First, we built models of MUTYH and *E. coli* MutY, either free or bound to DNA containing A·8oxoG, A·G, or A·C pairs (the five best models for each complex were considered). All protein models were supported by high pLDDT values (Supplementary Figure S1, Supplementary File S1) except for the disordered N- and C-terminal tails of MUTYH (residues 1–55 and 487–535), which were discarded in further modeling rounds, as well as the flexible interdomain linker of MUTYH and a short loop in the C-terminal domain of MutY. The high-confidence cores superimposed almost perfectly over the experimental structures of MUTYH and GsMutY (r.m.s.d. 0.5–0.7 Å for same-species and 1.5–1.7 Å for interspecies comparison). In all crystal structures of MUTYH and GsMutY bound to DNA with 8oxoG opposite the target nucleotide, Ser433 (MUTYH)/Ser308 (GsMutY) from the C-terminal NUDIX domain uniformly recognizes 8oxoG through hydrogen bonding to its N7 and O^8^ atoms (Oγ…H–N7 and N_backbone_–H…O^8^, respectively) [49,54]. In GsMutY, if G is present instead of 8oxoG, the Ser308 side chain is observed to rotate away (“rotamer 2”) and donate a hydrogen bond from its Oγ to a water molecule [74]. However, as G occupies almost the same position, rotamer 1 forming an Oγ–H…N7 bond would still be possible, even if less favorable energetically. In our AlphaFold models, both rotamers spontaneously appeared. In MUTYH, Ser433 remained in the rotamer 1 conformation in 5 out of 5 A·8oxoG models and 4/5 A·G models. However, rotamer 2 was prevalent in all 5 A·C models (Figure 7). The pyrimidine ring of C occupied the same position as the imidazole ring of 8oxoG/G but was not probed by Ser433, which instead was diverted to hydrogen-bond with His434. In *E. coli* MutY, the equivalent Ser295 better tolerated the A·C mismatch: it adopted the rotamer 1 configuration in 5/5 A·8oxoG models, 4/5 A·G models and 2/5 A·C models. The underlying structural feature seems to be that the +2 position from the 8oxoG-contacting Ser residue is occupied by Phe297 in *E. coli* MutY and Ile435 in MUTYH; stacking between Phe294 and Phe297 in the *E. coli* enzyme moves the His residue in the β-turn from the position of its human counterpart and offers more freedom for the Ser295 side chain rotation (Supplementary Figure S12). Ultimately, this might explain why MUTYH and *E. coli* MutY recognize A·G substrates with similar efficiency, but MUTYH is notably worse at processing A·C mispairs.

As other possible aberrant repair activities of MUTYH/MutY are concerned, neither protein was able to excise A from either position opposite a thymine dimer. As had been suggested earlier [34], this might be due to the rigid dimer preventing the insertion of the helix-invading wedge Tyr165(MUTYH)/Tyr82(EcoMutY) and the minor groove-penetrating motif ^125^QTQ^127^(MUTYH)/^42^QTQ^44^(EcoMutY) when the protein is bound in either register. Since AlphaFold 3 does not support thymine dimers, we have used pyDockDNA [50] to assess the possibility of the modeled MUTYH or *E. coli* MutY binding DNA with a thymine dimer in a correct orientation, starting with either the NMR structure of free AA·T = T-containing DNA (PDB ID 1TTD [51]) or the crystal structure of phage T4 endonuclease V, in which the 3′-A in an AA·T = T substrate is flipped out (1VAS [52]). While the docking faithfully recapitulated correct binding poses of DNA with an A·8oxoG pair, both before and after A eversion from the helix (Supplementary Figure S13A,B), neither MUTYH nor *E. coli* MutY approached the orientation that could be considered permissive for target A recognition in the AA·T = T substrates (Supplementary Figure S13C–E). The same was true when we docked DNA containing a CAC·GTG^Pt^ crosslink (NMR structures 1DA4, 1DA5, each containing two models [53]) (Supplementary Figure S13F–I). Nevertheless, we were able to generate MUTYH AlphaFold 3 models with the mismatched AAC·GTG (no crosslink) duplex of the same sequence used in our biochemical experiments (Supplementary Figure S1, cisPt25; Supplementary File S1), which showed A eversion, so the vicinity of the GTG trinucleotide could be inspected. While we observe slow excision of mismatched A by *E. coli* MutY (at least 1000-fold slower than from the A·8oxoG pair, cf. Figure 1C,D and Figure 5B) this might be due to the increased dynamics of T squeezed between the crosslinked G bases [53,78]. In a rare situation when MutY encounters a mismatched crosslinked AAC·GTG^Pt^ and manages to insert the wedge Tyr, this would push the thymine base into the major groove where it would be stacked against Trp255 in *E. coli* MutY. Human MUTYH, however, contains Leu384 in a homologous position, which is likely less stabilizing for the aromatic nucleobase than a planar indole moiety of Trp, compromising the enzyme’s activity (Figure 5A,B).

## 4. Discussion

Over the course of evolution, cells have developed DNA repair systems that can recognize damaged or abnormal bases amid an enormous excess of normal nucleobases in the genome [79]. However, faithful discrimination of miscoding base modifications is not always guaranteed; for example, oxidized dNTPs can be misincorporated opposite normal nucleotides in the template strand during DNA synthesis, creating mismatches that are difficult to repair [13]. Furthermore, under particular conditions, repair can be skewed: a regular base opposite modified residues may be removed, with the same regular nucleotide reinserted, and this cycle is repeated over again, leading to multiple rounds of unsuccessful repair [80]. Thus, futile base excision repair is at best useless and at worst mutagenic. It has been shown that bacterial (AlkA), yeast (MAG), and human (MPG/AAG/ANPG) alkylpurine-DNA glycosylases can remove normal adenine and guanine from regular DNA duplexes at physiologically significant rates [81]. Furthermore, bacterial and human nucleotide excision repair (NER) can initiate futile repair by targeting regular DNA and removing intact oligonucleotide fragments, which leads to futile cycles of excision and resynthesis [82]. More recently, we demonstrated sequence context-dependent futile excision of pyrimidines from undamaged DNA duplexes by human TDG [83]. It is thought that these futile excision activities of BER and NER proteins act preferentially on otherwise undamaged DNA; consequently, in quiescent cells, mutations induced by these activities would depend on the error rates of repair DNA polymerases [84]. Intriguingly, analysis of somatic mutation profiles in post-mitotic neurons and polyclonal smooth muscle cells revealed that neurons accumulate spontaneous mutations at a constant rate throughout life, even in the absence of cell division, at levels comparable to those observed in mitotically active tissues [85,86]. These data imply that spontaneous mutations occurring in the absence of cell division and DNA replication could contribute significantly to somatic mutagenesis.

It appears that in the absence of mismatch repair, in the *E. coli mutS* strain or in the absence of the oxidized nucleotide pool-cleansing enzyme in the *E. coli mutT* strain, the remaining component of the GO pathway, MutY protein, could play a mutagenic role by enhancing the rate of A→G and A→C mutations, respectively [13,87]. Concerning mammalian adenine-DNA glycosylases, the loss of MUTYH in the mismatch repair-deficient context (*Msh2^-/-^*) reduces cancer progression in mice [25] and humans [88], suggesting that MUTYH may participate in some aberrant repair pathway that promotes cancer. Intriguingly, the loss of MUTYH was associated with enhanced survival to 6-thioguanine and UVA radiation-induced DNA damage, alkylating agent N-methyl-N’-nitro-N-nitrosoguanidine (MNNG), mitomycin C and UV radiation (reviewed in [20]). Taken together, these observations suggest that MUTYH is involved in DNA damage signaling by sensitizing cells to genotoxic agents that may help eliminate cells harboring excess damage to avoid mutations. Previously, we hypothesized that, like human TDG, adenine-DNA glycosylases could act aberrantly by removing regular adenine when opposite modified bases other than 8oxoG. This in turn would create highly cytotoxic and mutagenic bi-stranded DNA damage consisting of a single-strand break opposite an unrepaired lesion, which would be difficult to repair [89]. This type of lesion with persistent a SSB would strongly activate PARP1 and ATR/Chk1 signaling and may lead to both apoptosis and senescence, depending on the cellular context and type of DNA damage. In the present study, we systematically examined the DNA glycosylase activities of MutY and MUTYH for the presence of aberrant excision of adenine in damaged DNA duplexes.

In our earlier study, we demonstrated that the *E. coli* MutY protein does not excise A residues in DNA duplexes containing UV-induced CPD and 6-4PP adducts. Furthermore, the study of UV-induced mutagenesis in BER/NER-deficient *E. coli* strains revealed that the induction of C·G→T·A transitions after UV exposure does not depend on MutY, thus ruling out the role of a putative aberrant BER in vivo [34]. In the present work, we successfully purified active recombinant human adenine-DNA glycosylase, which made it possible to examine the enzymatic activities of MUTYH and bacterial counterpart towards non-canonical DNA substrates.

The purified bacterial MutY and human recombinant MUTYH proteins exhibited excellent activity towards their classic A·8oxoG substrate (Figure 1), indicating that our enzyme preparations can be used to examine DNA glycosylase activity on non-classical DNA substrates. Using our highly active enzyme preparations, we confirmed previous observations showing that MutY and MUTYH are active on A·G and A·C substrates, with the bacterial protein being more active compared to the human counterpart (Figure 2A). In agreement with previous study, MUTYH was more active towards 2oxoA·G duplexes than MutY (Figure 2C). Thus, our purified MutY and MUTYH passed the necessary controls before using them on non-characterized DNA substrates.

Here, we report that the human MUTYH and *E. coli* MutY proteins do not remove A residues opposite common endogenous base modifications such as uracil, oxidized cytosine, ethenobases, alpha-anomeric deoxyadenosine and synthetic AP sites, indicating that during evolution, selection pressure optimized these mismatch-specific DNA glycosylases for high efficiency and specificity (Figure 3A,B). Although the A·8oxoG mispair is the most preferred DNA substrate for *E. coli* and human adenine-DNA glycosylases, it has been demonstrated that MutY and MUTYH excise A when opposite C8-modified guanine derivatives, including the 8brG residue [45,64,65]. Here, we show for the first time that MutY and MUTYH excise adenine when opposite 8meG and 8moG residues in duplex DNA (Figure 3C). Interestingly, MutY exhibits relatively good efficiency towards A*·8moG, A*·8brG and A*·8meG duplexes, and MUTYH towards A*·8brG and A*·8moG that is comparable to that of A*·G duplex, suggesting a possible role of these activities in mutation prevention in vivo (Table 1).

We further demonstrated that both adenine-DNA glycosylases do not remove A residues opposite bulky UV-induced CPD and 6-4PP adducts in the short duplex oligonucleotides even after 18 h of incubation at 37 °C (Figure 4). Importantly, in the same DNA sequence context used for UV lesions, MUTYH and MutY efficiently excise mismatched A residues when opposite G (Figure 4). These biochemical results suggest that human adenine-DNA glycosylase does not participate in the repair of UV-induced DNA damage in human cells. The incapacity of MUTYH to recognize A opposite CPD and 6-4PP could be due, at least in part, to a severe topological constraint caused by the covalent bonds between two damaged pyrimidines: the distance between the two thymines in the dimer is smaller than that required for accommodation within the active pocket of MutY and cannot be increased. Thus, the pre-catalytic complex formed between MUTYH and DNA containing a UV-induced dimer would be disrupted, leading to a dramatic decrease in the efficiency of the reaction.

MUTYH and MutY also do not excise adenine opposite bulky 8,5′-*cyclo*-2′-deoxyadenosine (Figure 5C). Nevertheless, upon a long incubation time (18 h), we observed MutY-catalyzed aberrant excision of an adenine residue opposite bulky guanine adducts such as 1,3-d(GpNpG) cisplatin intra-strand crosslink (Figure 5A,B). This result suggests that MutY may prevent misincorporation of adenine opposite bulky guanine lesions during DNA translesion synthesis bypass in *E. coli*. Although this aberrant activity of MutY is very slow and inefficient, it may play an anti-mutagenic role in SOS-induced stationary phase cells. While the aberrant activity of any enzyme is, by definition, much lower than its classical, physiologically relevant activity, the structural reasons for the observed non-canonical activities of a given enzyme may shed light on hidden biological functions or on the molecular mechanism of trade-off between fidelity and efficiency of that enzyme. For MUTYH/MutY, the physiological substrate is believed to be A·8oxoG, and the origins of their activity on the A·G, A·C and other non-canonical substrates may reveal some novel aspects of mutagenesis in the living cell. Our modeling exercises consistently support the biochemical findings that MUTYH exhibits more stringent DNA substrate specificity than MutY: lower activities of the human enzyme on the A·C and AAC·GTG^Pt^ substrates compared with its *E. coli* counterpart find possible explanations in the structural features of both enzymes, such as the presence of the aliphatic Leu384 and Ile435 in MUTYH instead of the aromatic Trp and Phe in the homologous positions of *E. coli* MutY. It should be noted, however, that *E. coli* MutY seems to be more diverged from both MUTYH and GsMutY in some important functional regions, including not only these positions (both Leu in GsMutY), but also several amino acids interacting with adenine in the binding pocket and the mechanistically important Tyr204(MUTYH)/Tyr127(GsMutY), which is replaced with Ser in the *E. coli* protein. It may well be that the biological role of MutY in different species is flexible and depends on the dominating genotoxic factors that affect the spectrum of the lesions generated in DNA, counteracting negative selection against aberrant activities.

Based on the recently reported structure of MUTYH [49], we have proposed that several naturally occurring mutations in the *MUTYH* gene (natural germline polymorphisms or somatic mutations) could produce a protein with impaired activity, possibly bearing cancer risks (Supplementary Figure S14). The G169D variant is rare in natural populations: its allele frequency is 9.12 × 10^−6^ in the Million Exome Variants database [90]; it is reported in cancers as a germline mutation, more commonly from Eastern European populations [91,92] (Poland, Russia). The variant appears in the saturation mapping functional screen [93] as “Likely pathogenic”. Gly169 is not exceptionally conserved (Supplementary Figure S2E), but lies next to Arg168, which occupies the DNA groove near 8oxoG; the R168H mutant is inactive [94]. Changing Gly169 to a negatively charged Asp can divert Arg168 from its position, so it was expected that MUTYH G169D would be less active than the wild-type protein. Indeed, we found its catalytic activity, as characterized by the *k*_obs_ value, is ~5-fold lower compared with wild-type MUTYH. Another variant that caught our attention was G202E, also rare in general populations (allele frequency 3.72 × 10^−6^ in gnomAD [95], 6.08 × 10^−6^ in Million Exome Variants), but amply reported in cancer patients as a germline mutation with conflicting pathogenicity assignments [96,97,98,99]. Gly202 occupies the last position in the absolutely conserved GVG motif in the hairpin of the DNA-binding helix–hairpin–helix (Supplementary Figure S2C), making a turn near the DNA backbone at the 2nd nucleotide 3′ from the target A. We also expected this variant to be non-functional, and found the activity reduced nearly tenfold. These results highlight the usefulness of a structure-guided approach to VUS classification, which may significantly improve the prognostic ability of present-day algorithms, mostly based on residue conservation patterns [100]. Furthermore, we demonstrated that MUTYH variants tested do not exhibit increased aberrant activities towards non-canonical DNA substrates, suggesting that cancer risk is mainly associated with the decreased repair of A*·8oxoG in genome.

## 5. Conclusions

Mismatch-specific DNA glycosylases that remove regular DNA bases are intrinsically prone to aberrant activity because they lack specific mechanisms for distinguishing the template strand from the damaged strand. Multiple lines of evidence indicate that, under certain conditions, the presence of MutY and MUTYH can increase cellular sensitivity and mutagenesis in response to genotoxic stress, especially in DNA repair-deficient cells, suggesting that these DNA glycosylases may act aberrantly on unrepaired, persistent DNA damage. To examine this possibility, we investigated the activity of human and bacterial adenine-DNA glycosylases on non-canonical DNA substrates in which a normal adenine residue was placed opposite various base modifications. Our data show that, unlike MutY, MUTYH is not prone to erroneous excision of regular adenine residues from damaged DNA duplexes. We hypothesize that the human enzyme exerts its genotoxic effects through DNA damage signaling pathways involving complex interactions with genome integrity maintenance systems. In addition, we propose that bacterial MutY is more prone to aberrant excision of normal adenines and may participate in DNA damage tolerance pathways.

## Figures and Tables

**Table 2:** Pre-steady-state kinetics of adenine excision by human MUTYH and *E. coli* MutY from lesion-containing DNA duplexes.

Substrate	MUTYH, *k*obs (min^−1^)	*k*_obs[A·8oxoG]_/*k*_obs[A·X]_	MutY, *k*obs (min^−1^)	*k*_obs[A·8oxoG]_/*k*_obs[A·X]_
A*·8oxoG	0.8 ± 0.1 ^a^	1	4.3 ± 0.03	1
A*·G	0.10 ± 0.01	8	0.14 ± 0.02	31
A*·8meG	n.d. ^b^		0.01 ± 0.003	430
A*·8moG	0.18 ± 0.05	4.4	0.09 ± 0.02	48
A*·8brG	0.10 ± 0.01	8	0.12 ± 0.01	36
AAC*·GTG^Pt^	n.d.		0.04 ± 0.02	108

^a^ Values are reported as mean ± SD. ^b^ n.d. = not determined.

**Table 3:** Observed rate constants for adenine excision by wild-type and variant MUTYH proteins on the A*·8oxoG substrate.

Enzyme	*k*obs (min^−1^)	*k*_obs[WT]_/*k*_obs[Mutant]_
MUTYH-WT	3.2 ± 0.8	1
MUTYH-G202E	0.34 ± 0.12	9.4
MUTYH-G169D	0.83 ± 0.28	3.9
MUTYH-Y165C	0.091 ± 0.013	35

## Data Availability

The original contributions presented in this study are included in the article/Supplementary Material. Further inquiries can be directed to the corresponding authors.

## Supplementary Materials

The following supporting information can be downloaded at https://www.mdpi.com/article/10.3390/biom16081214/s1, Supplementary File S1: AlphaFold3 generated models of full-length human MUTYH and *E. coli* MutY, either free or bound to various mismatch-containing DNA. Supplementary File S2: Conservation of key residues in MutY homologs. Figure S1: Aggregated pLDDT plots for models of MUTYH or EcoMutY and their complexes with DNA. Figure S2: Graphs of conservation around key A- and 8-oxoG-recognizing residues. Figure S3: Time-dependent adenine excision in A·8oxoG duplex; Figure S4: Time-dependent adenine excision in A·G duplex; Figure S5: Time-dependent adenine excision in A·8meG duplex; Figure S6: Time-dependent adenine excision in A·8moG duplex; Figure S7: Time-dependent adenine excision in A·8brG duplex; Figure S8: Numeration of MUTYH residues used in the literature; Figure S9: SDS-PAGE of the purified MUTYH proteins; Figure S10: Time-dependent cleavage of A·8oxoG duplex by MUTYH varinats; Figure S11: Activities of MUTYH variants on non-canonical DNA substrates; Figure S11: Activities of wild-type and mutant variants of MUTYH; Figure S12: Structure of the tip of the β-hairpin interacting with the orphaned nucleotide in MUTYH bound to A·8oxoG; Figure S13: Examples of distributions of the 100 highest-scoring docking poses generated by pyDockDNA; Figure S14: Positions of Gly169 and Gly202 in the MUTYH–DNA structure (PDB ID 8FAY). References [101,102] are cited in the Supplementary Materials.

## Abbreviations

The following abbreviations are used in this manuscript:

CPD	Cyclobutane pyrimidine dimers
6-4PP	Pyrimidine (6-4) pyrimidone photoproduct
cdA	8,5′-*cyclo*-2′-deoxyadenosine
GNG^Pt^	1,3-d(GpNpG) cisplatin intra-strand crosslink
8oxoG	7,8-dihydro-8-oxoguanine
2oxoA	1,2-dihydro-2-oxoadenine
AP	Apurinic/apyrimidinic site
THF	Synthetic AP site
5ohC	5-hydroxycytosine
εC	3,*N*^4^-ethenocytosine
εA	1,*N*^6^-ethenoadenine
αdA	Alpha-anomeric 2′-deoxyadenosine
8meG	8-methylguanine
8moG	8-methoxyguanine
8brG	8-bromoguanine
MutY	*Escherichia coli* adenine-DNA glycosylase
AlkA	*Escherichia coli* 3-methyladenine-DNA glycosylase II
Fpg	*Escherichia coli* Fapy-DNA glycosylase
MUTYH	Human adenine-DNA glycosylase
ANPG	Human alkyl-N-purine-DNA glycosylase
BER	Base excision repair
NER	Nucleotide excision repair
