# Supplementary material for: DNA Damage Recognition by Bacterial and Human Adenine-DNA Glycosylases: Insights from Non-Canonical Substrates
Source: Biomolecules. 2026 Aug 20;16(8):1214. doi: 10.3390/biom16081214 (PMC13511376; doi:10.3390/biom16081214)
Supplement: Supplementary file 1 [file biomolecules-16-01214-s001.zip › Supplementary Figures S1-S14_Biomolecules_revised2_final.pdf]

Article

DNA Damage Recognition by Bacterial and Human Adenine-DNA Glycosylases: Insights from Non-Canonical Substrates

Ulan Sarsenbayeva [1,2], Didier Gasparutto [3], Nicolas Geacintov [4], Alexander A. Ishchenko [5], Gulzhan Zhamanbayeva [1], Kamalidin O. Sharipov [2,6], Carlos H. Trasviña-Arenas [7], Sheila S. David [8], Dmitry O. Zharkov [9,10], Bakhyt T. Matkarimov [11,12], Murat Saparbaev [1,5,*] and Sabira Taipakova [1,13,*]

[1] Faculty of Biology and Biotechnology, Al-Farabi Kazakh National University, Almaty 050040, Kazakhstan; ulansarsenbayeva@gmail.com (U.S.); gulzhan.kaznu.kz@gmail.com (G.Z.)
[2] Department of Biochemistry, Asfendiyarov Kazakh National Medical University, Almaty 050012, Kazakhstan; sharipov.k@kaznmu.kz
[3] CEA, CNRS, Institut National Polytechnique de Grenoble, Institut de Recherche Interdisciplinaire de Grenoble, Systèmes Moléculaires et nanoMatériaux pour l'Énergie et la Santé—Unité Mixte de Recherche 5819, University Grenoble Alpes, 38000 Grenoble, France; didier.gasparutto@cea.fr
[4] Chemistry Department, New York University, 31 Washington Place, New York, NY 10003, USA; ng1@nyu.edu
[5] CNRS-UMR9019 Genome Integrity and Cancers, Gustave Roussy Cancer Campus, Université Paris-Saclay, 94805 Villejuif, France; alexander.ishchenko@gustaveroussy.fr
[6] Structural and Functional Genomics Laboratory, M.A. Aitkhozhin Institute of Molecular Biology and Biochemistry, Almaty 050012, Kazakhstan
[7] Centro de Investigación sobre el Envejecimiento, Centro de Investigación y de Estudios Avanzados (CINVESTAV), Unidad Sede Sur, Tlalpan, Ciudad de México 14330, Mexico; carlos.trasvina@cinvestav.mx
[8] Chemical Biology Graduate Program, Department of Chemistry and Chemistry, University of California, Davis, CA 95616, USA; ssdavid@ucdavis.edu
[9] SB RAS Institute of Chemical Biology and Fundamental Medicine, Lavrentiev Av. 8, Novosibirsk 630090, Russia; oxoguanine@gmail.com
[10] Department of Natural Sciences, Novosibirsk State University, 2 Pirogova St., Novosibirsk 630090, Russia
[11] National Laboratory Astana, Nazarbayev University, Astana 010000, Kazakhstan; bmatkarimov@nu.edu.kz
[12] L.N. Gumilyov Eurasian National University, Astana 010000, Kazakhstan
[13] Scientific Research Institute of Biology and Biotechnology Problems, Al-Farabi Kazakh National University, Almaty 050040, Kazakhstan
* Correspondence: murat.saparbaev@gustaveroussy.fr (M.S.); sabira.taipakova@gmail.com (S.T.); Tel.: +33-(1)-42115404 (M.S.); +7-(727)-3773334 (S.T.)

Supplementary Figures S1 – S14

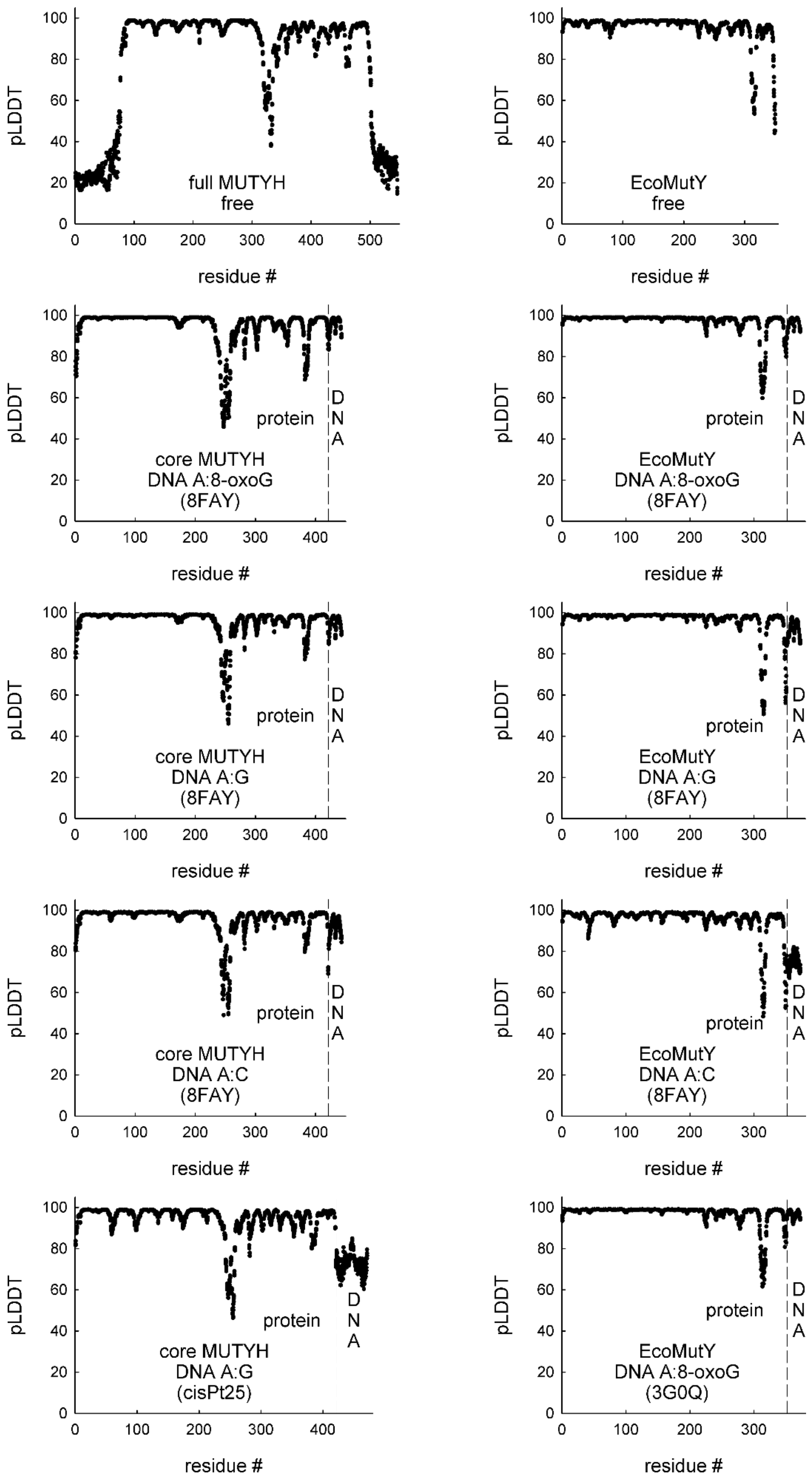

pLDDT
residue #
full MUTYH
free
EcoMutY
free
protein
DNA
core MUTYH
DNA A:8-oxoG
(8FAY)
EcoMutY
DNA A:8-oxoG
(8FAY)
core MUTYH
DNA A:G
(8FAY)
EcoMutY
DNA A:G
(8FAY)
core MUTYH
DNA A:C
(8FAY)
EcoMutY
DNA A:C
(8FAY)
core MUTYH
DNA A:G
(cisPt25)
EcoMutY
DNA A:8-oxoG
(3G0Q)

Figure S1. Aggregated pLDDT plots for models of MUTYH or EcoMutY and their complexes with DNA. Cα/C1′ pLDDT values for five best models are shown in each plot. The nature of the protein and DNA are indicated on the plots. Core MUTYH, the fragment comprising residues 56–486 (numeration after [A]).

[73]. Slupska M.M., Baikalov C., Luther W.M., Chiang J.-H., Wei Y.-F., Miller J.H. (1996) Cloning and sequencing a human homolog (*hMYH*) of the *Escherichia coli mutY* gene whose function is required for the repair of oxidative DNA damage. *J. Bacteriol.*, **178**, p. 3885-3892.

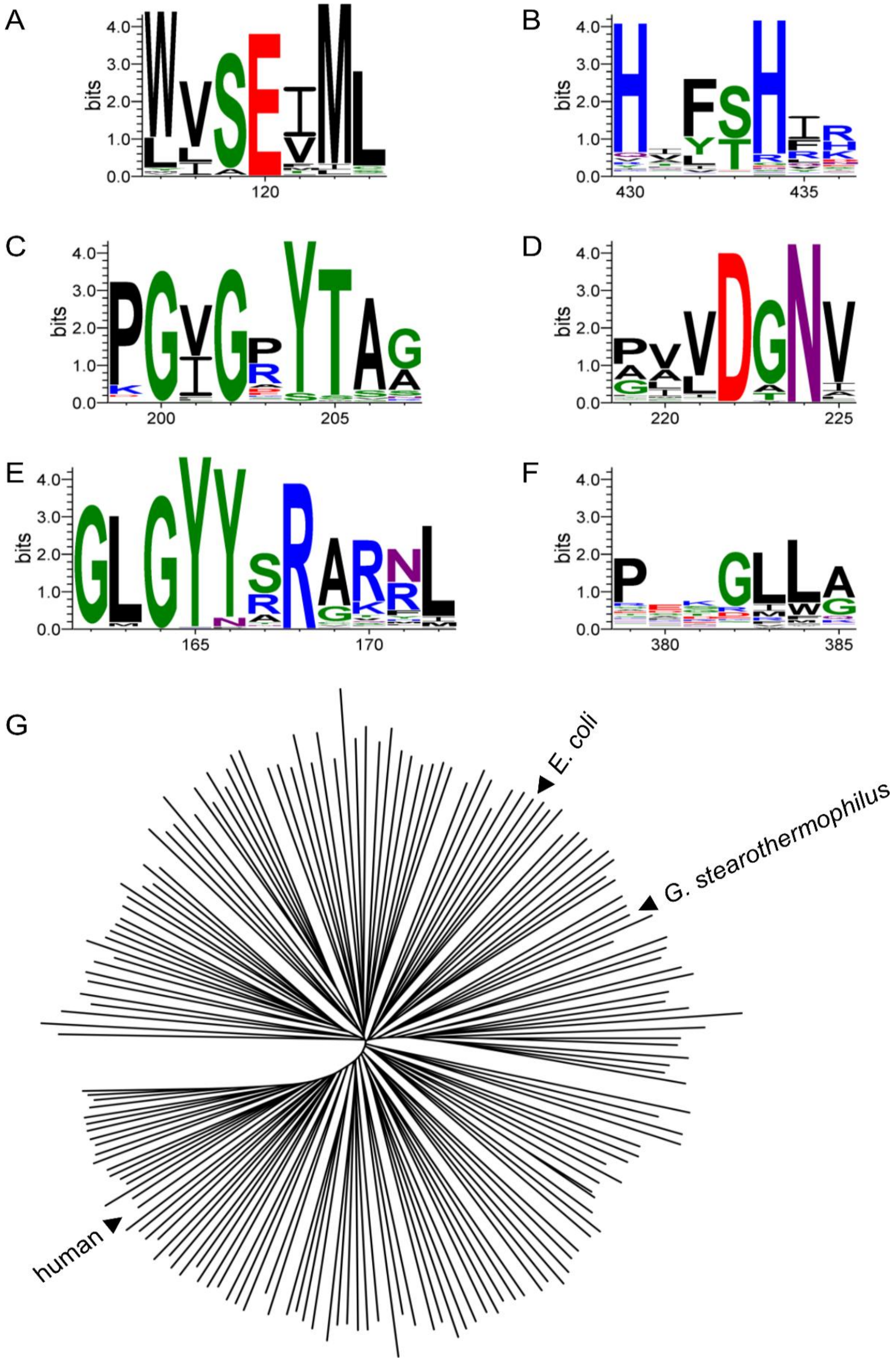

Figure S2. (A–F), graphs of conservation around key A- and 8-oxoG-recognizing residues (Glu120 and Ser433, respectively; (**A,B**), catalytic residues (Tyr204 and Asp222; (**C,D**), two residues affected by the known founder mutations in MUTYH-associated cancers (Tyr165 and Gly382; (**E,F**), and two residues affected by the cancer-

derived mutations investigated in this work (Gly202 in C and Gly169 in **E**). The graphs were produced with WebLogo v3.9 [B]. The amino acids are color-coded as polar (green), neutral (purple), basic (blue), acidic (red) and hydrophobic (black). G, unrooted tree of 169 MutY homologs from eukaryotes, bacteria, and archaea. The tree was built with iTOL v7 [C].

[55]. Crooks, G.E.; Hon, G.; Chandonia, J.M.; Brenner, S.E. WebLogo: A sequence logo generator. *Genome Res.* **2004**, *14*, 1188–1190. https://doi.org/10.1101/gr.849004.
[101]. Letunic, I.; Bork, P. Interactive Tree of Life (iTOL) v6: recent updates to the phylogenetic tree display and annotation tool. Nucleic Acids Res 2024, 52, W78-w82, https://doi.org/10.1093/nar/gkae268.

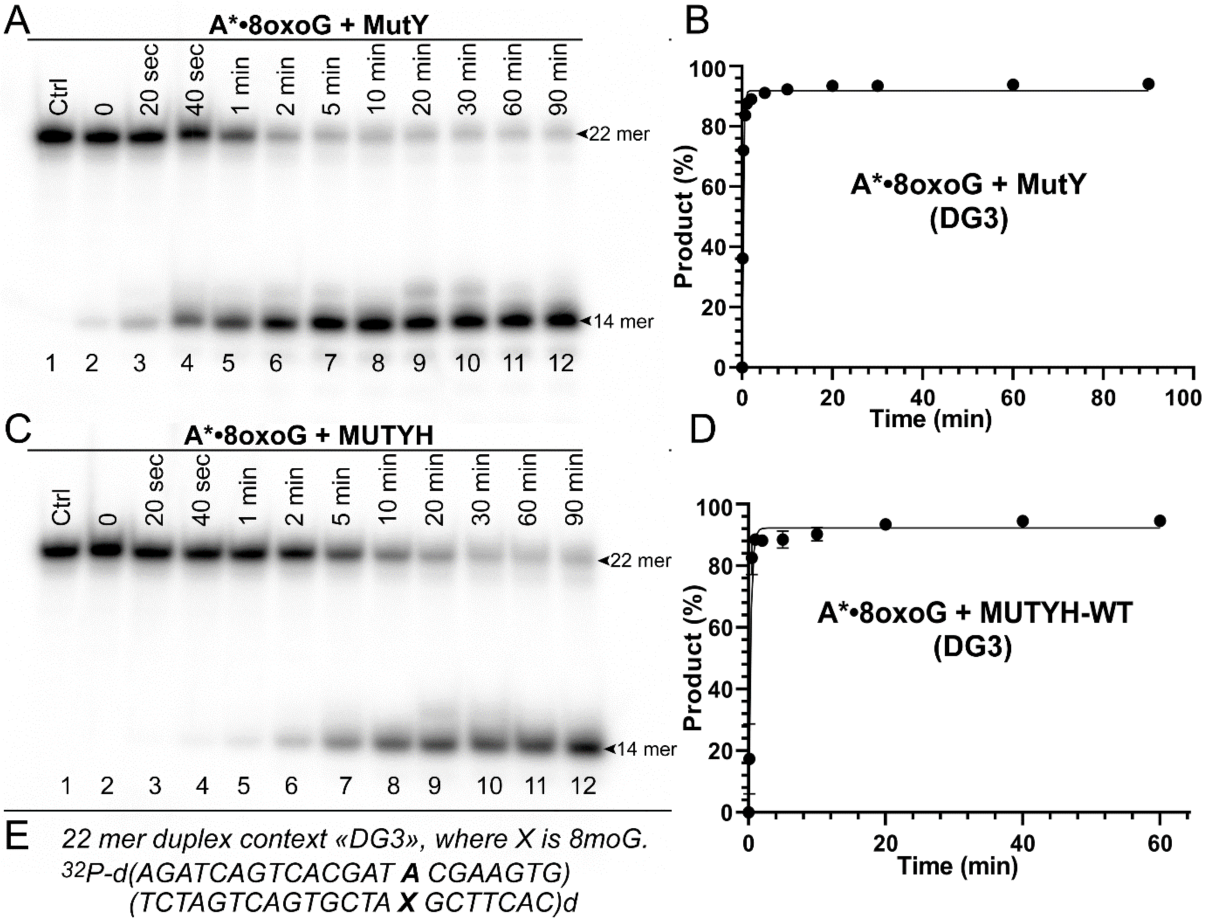

Figure S3. Time-dependent adenine excision in A•8oxoG oligonucleotde duplex (context "**DG1**") by *E. coli* MutY (**A,B**) and MUTYH (**C,D**). 20 nM of 22 mer 5′-^{32}P-labelled A*•8oxoG duplex oligonucleotide was incubated with under single turnover conditions; 200 nM of MUTYH or MutY proteins for varying period of time at 37°C. Arrows indicate the sizes of the DNA substrate and cleavage fragments. (**B,D**) Plots of pre-steady-state single turnover kinetic of MutY- and MUTYH-catalysed cleavage of 22 mer A•8oxoG duplexes. Mean ± SD of three independent experiments is shown. (**E**) Schematic representation of 22 mer duplex sequence with target adenine residue and opposite modified base. For details see Materials and Methods section.

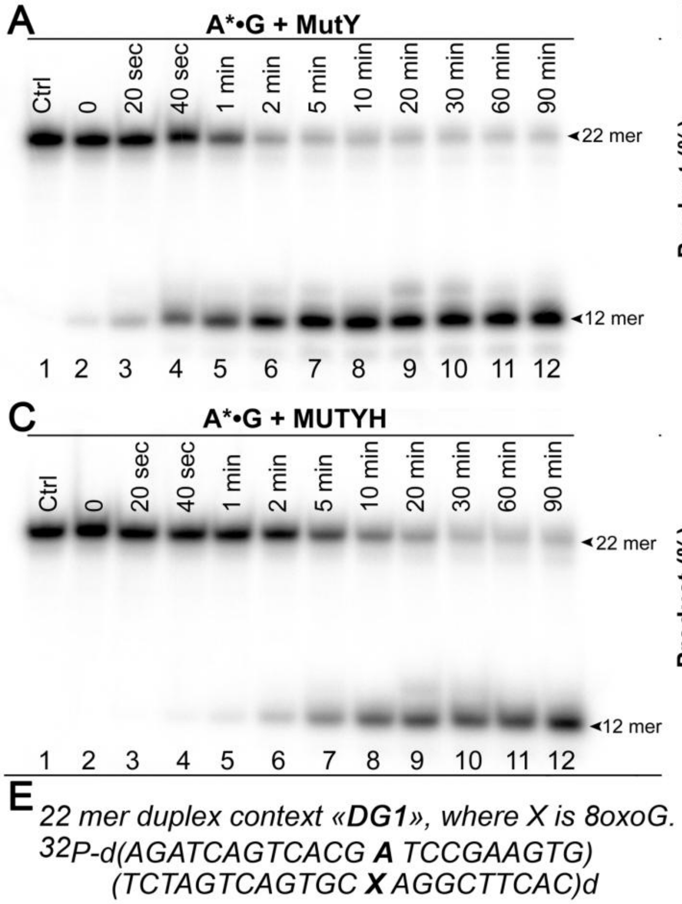

Figure S4. Time-dependent adenine excision in A•G oligonucleotde duplex (context "**DG1**") by *E. coli* MutY (**A,B**) and MUTYH (**C,D**). 20 nM of 22 mer 5′-^{32}P-labelled A*•G duplex oligonucleotide was incubated with under single turnover conditions; 200 nM of MUTYH or MutY proteins for varying period of time at 37°C. Arrows indicate the sizes of the DNA substrate and cleavage fragments. (**B,D**) Plots of pre-steady-state single turnover kinetic of MutY- and MUTYH-catalysed cleavage of 22 mer A•G duplexes. Mean ± SD of three independent experiments is shown. (**E**) Schematic representation of 22 mer duplex sequence with target adenine residue and opposite modified base. For details see Materials and Methods section.

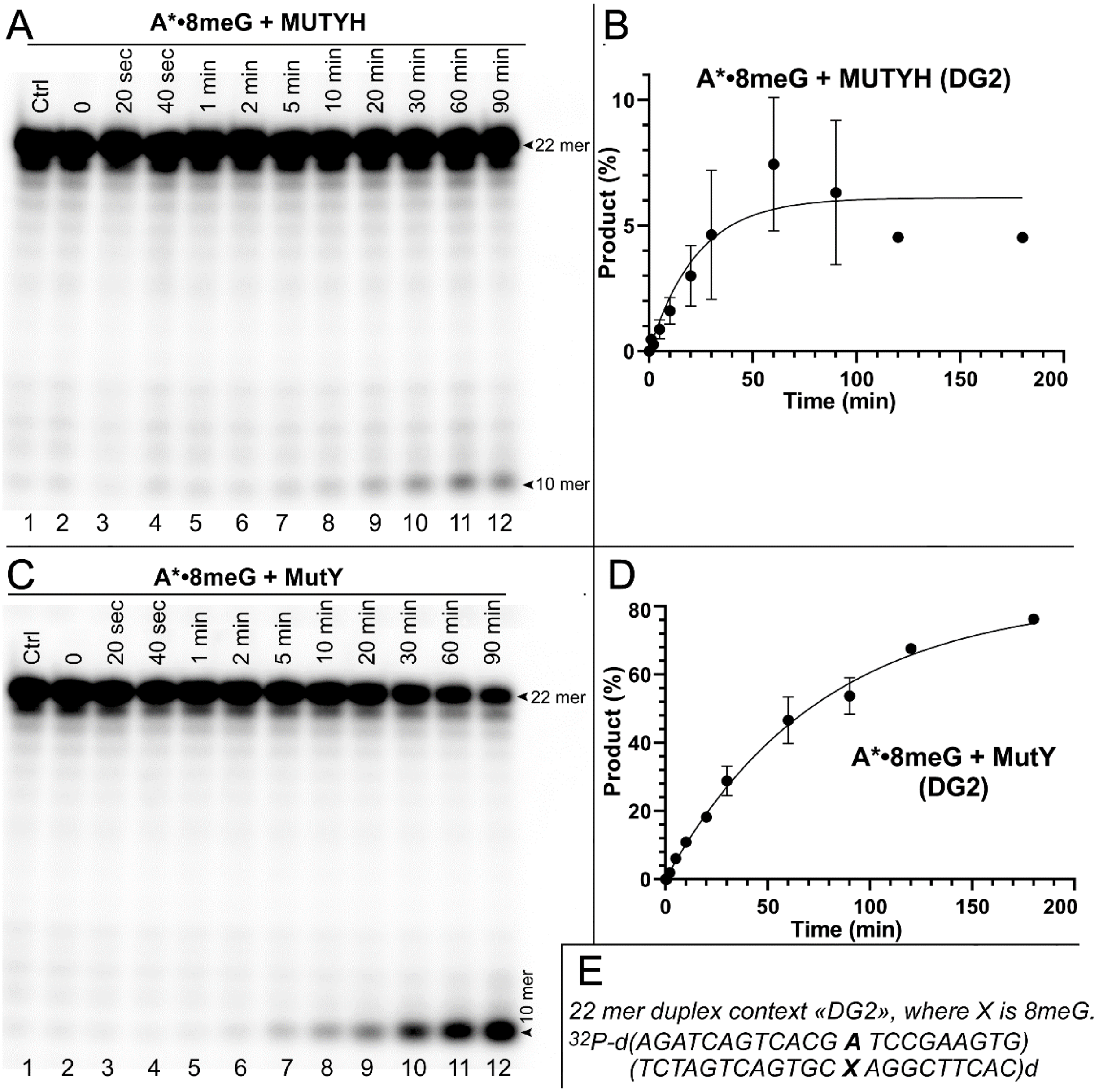

Figure S5. Time-dependent adenine excision in A•8meG oligonucleotde duplex (context "**DG2**") by MUTYH (**A**,**B**) and *E. coli* MutY (**C**,**D**). 20 nM of 22 mer 5′-^{32}P-labelled A*•8meG duplex oligonucleotide was incubated with under single turnover conditions; 200 nM of MUTYH or MutY proteins for varying period of time at 37°C. Arrows indicate the sizes of the DNA substrate and cleavage fragments. (**B**,**D**) Plots of pre-steady-state single turnover kinetic of MUTYH- and MutY-catalysed cleavage of 22 mer A•8meG duplexes. Mean ± SD of three independent experiments is shown. (**E**) Schematic representation of 22 mer duplex sequence with target adenine residue and opposite modified base. For details see Materials and Methods section.

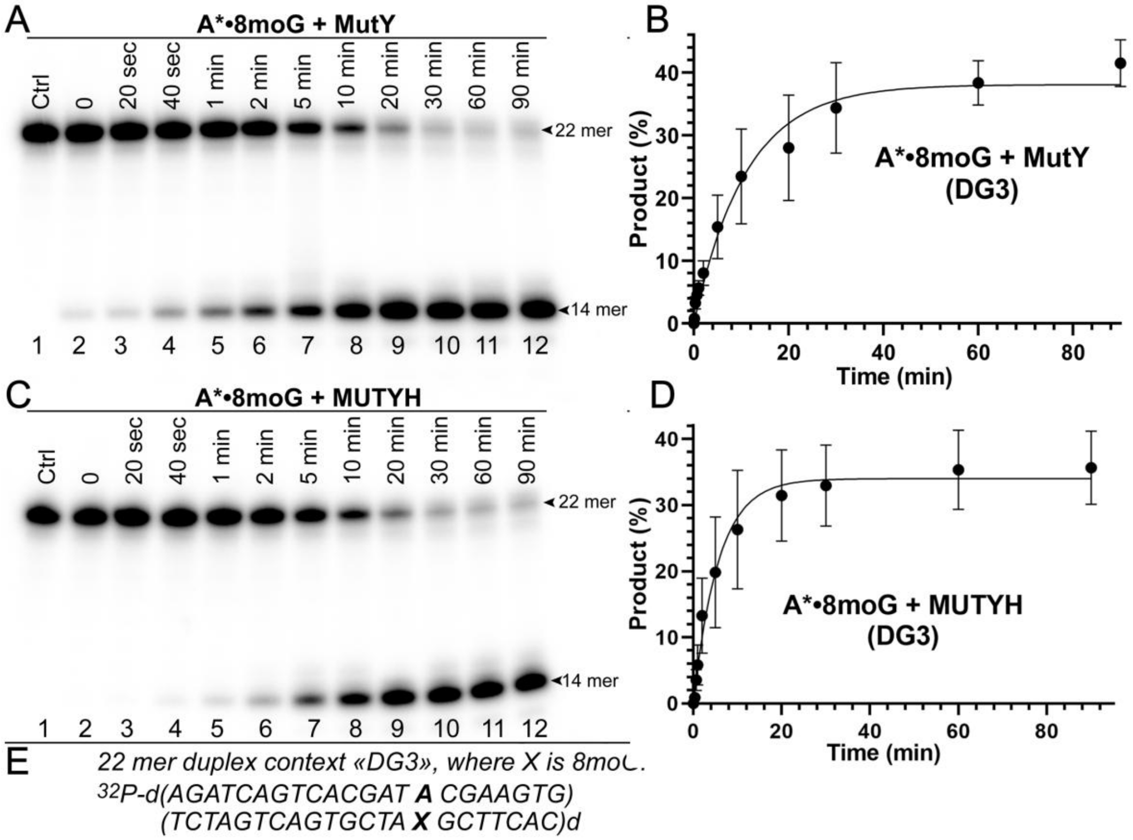

Figure S6. Time-dependent adenine excision in A•8moG oligonucleotde duplex (context "**DG3**") by *E. coli* MutY (**A,B**) and MUTYH (**C,D**). 20 nM of 22 mer 5′-^{32}P-labelled A*•8moG duplex oligonucleotide was incubated with under single turnover conditions; 200 nM of MUTYH or MutY proteins for varying period of time at 37°C. Arrows indicate the sizes of the DNA substrate and cleavage fragments. (**B,D**) Plots of pre-steady-state single turnover kinetic of MutY- and MUTYH-catalysed cleavage of 22 mer A•8moG duplexes. Mean ± SD of three independent experiments is shown. (**E**) Schematic representation of 22 mer duplex sequence with target adenine residue and opposite modified base. For details see Materials and Methods section.

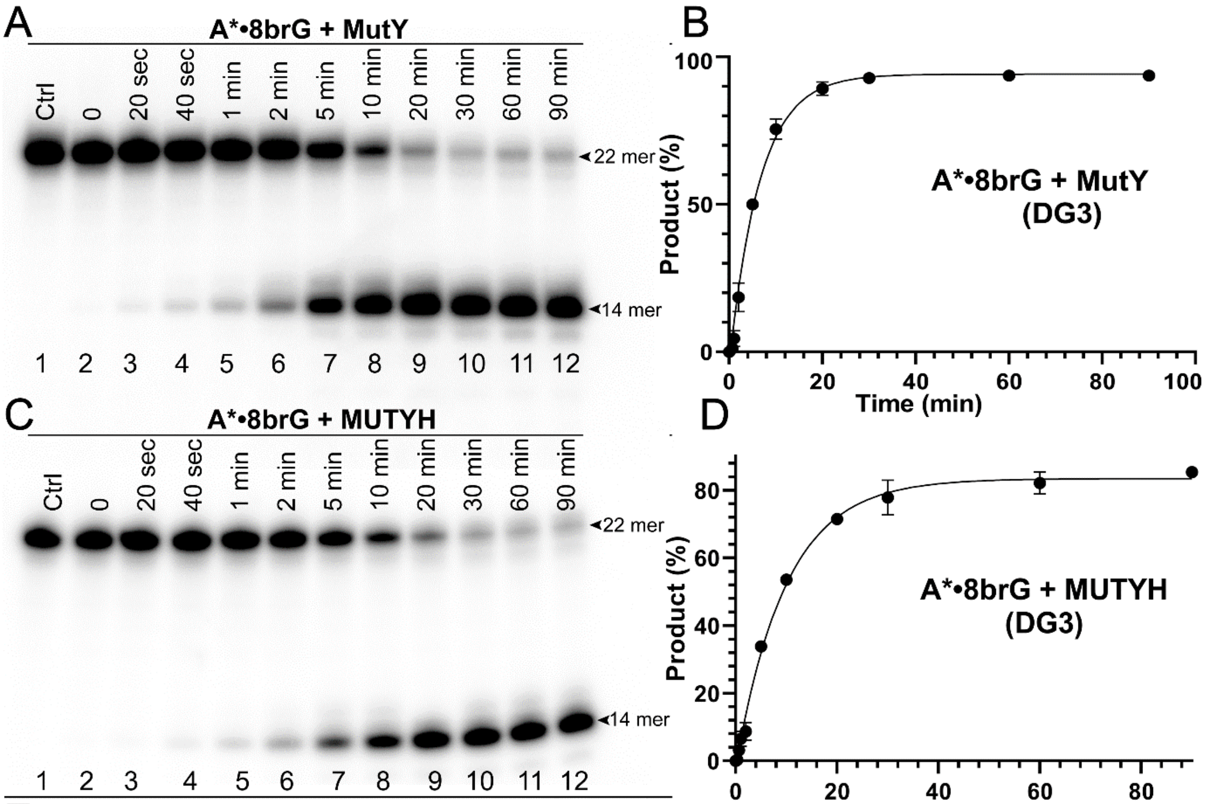

E *22 mer duplex context «DG3», where X is 8brG.*
*^{32}P-d(AGATCAGTCACGAT **A** CGAAGTG)*
*(TCTAGTCAGTGCTA **X** GCTTCAC)d*

Figure S7. Time-dependent adenine excision in A•8brG oligonucleotde duplex (context "**DG3**") by *E. coli* MutY (**A,B**) and MUTYH (**C,D**). 20 nM of 22 mer 5′-^{32}P-labelled A*•8brG duplex oligonucleotide was incubated with under single turnover conditions; 200 nM of MUTYH or MutY proteins for varying period of time at 37°C. Arrows indicate the sizes of the DNA substrate and cleavage fragments. (**B,D**) Plots of pre-steady-state single turnover kinetic of MutY- and MUTYH-catalysed cleavage of 22 mer A•8brG duplexes. Mean ± SD of three independent experiments is shown. (**E**) Schematic representation of 22 mer duplex sequence with target adenine residue and opposite modified base. For details see Materials and Methods section.

```
[1]    MTPLVSRLSRLWAIMRKPRAAVGSGHRKQAASQEGRQKHAKNNSQAKPSACDG------- 53
[2]    ----------------------------------------------------[60]…GHM 63
[3]    MTPLVSRLSRLWAIMRKPRAAVGSGHRKQAASQEGRQKHAKNNSQAKPSACDGMIAECPG 60
[4]    --------------MRKPRAAVGSGHRKQAASQEGRQKHAKNNSQAKPSACDG------- 39

[1]    ----LARQPEEVVLQASVSSYHLFRDVAEVTAFRGSLLSWYDQEKRDLPWRRRAEDEMDL 109
[2]    ACDGLARQPEEVVLQASVSSYHLFRDVAEVTAFRGSLLSWYDQEKRDLPWRRRAEDEMDL 123
[3]    APAGLARQPEEVVLQASVSSYHLFRDVAEVTAFRGSLLSWYDQEKRDLPWRRRAEDEMDL 120
[4]    ----LARQPEEVVLQASVSSYHLFRDVAEVTAFRGSLLSWYDQEKRDLPWRRRAEDEMDL 95

[1]    DRRAYAVWVSEVMLQQTQVATVINYYTGWMQKWPTLQDLASASLEEVNQLWAGLGYYSRG 169
[2]    DRRAYAVWVSEVMLQQTQVATVINYYTGWMQKWPTLQDLASASLEEVNQLWAGLGYYSRG 183
[3]    DRRAYAVWVSEVMLQQTQVATVINYYTGWMQKWPTLQDLASASLEEVNQLWAGLGYYSRG 180
[4]    DRRAYAVWVSEVMLQQTQVATVINYYTGWMQKWPTLQDLASASLEEVNQLWAGLGYYSRG 155

[1]    RRLQEGARKVVEELGGHMPRTAETLQQLLPGVGRYTAGAIASIAFGQATGVVDGNVARVL 229
[2]    RRLQEGARKVVEELGGHMPRTAETLQQLLPGVGRYTAGAIASIAFGQATGVVDGNVARVL 243
[3]    RRLQEGARKVVEELGGHMPRTAETLQQLLPGVGRYTAGAIASIAFGQATGVVDGNVARVL 240
[4]    RRLQEGARKVVEELGGHMPRTAETLQQLLPGVGRYTAGAIASIAFGQATGVVDGNVARVL 215

[1]    CRVRAIGADPSSTLVSQQLWGLAQQLVDPARPGDFNQAAMELGATVCTPQRPLCSQCPVE 289
[2]    CRVRAIGADPSSTLVSQQLWGLAQQLVDPARPGDFNQAAMELGATVCTPQRPLCSQCPVE 303
[3]    CRVRAIGADPSSTLVSQQLWGLAQQLVDPARPGDFNQAAMELGATVCTPQRPLCSQCPVE 300
[4]    CRVRAIGADPSSTLVSQQLWGLAQQLVDPARPGDFNQAAMELGATVCTPQRPLCSQCPVE 275

[1]    SLCRARQRVEQEQLLASGSLSGSPDVEECAPNTGQCHLCLPPSEPWDQTLGVVNFPRKAS 349
[2]    SLCRARQRVEQEQLLASGSLSGSPDVEECAPNTGQCHLCLPPSEPWDQTLGVVNFPRKAS 363
[3]    SLCRARQRVEQEQLLASGSLSGSPDVEECAPNTGQCHLCLPPSEPWDQTLGVVNFPRKAS 360
[4]    SLCRARQRVEQEQLLASGSLSGSPDVEECAPNTGQCHLCLPPSEPWDQTLGVVNFPRKAS 335

[1]    RKPPREESSATCVLEQPGALGAQILLVQRPNSGLLAGLWEFPSVTWEPSEQLQRKALLQE 409
[2]    RKPPREESSATCVLEQPGALGAQILLVQRPNSGLLAGLWEFPSVTWEPSEQLQRKALLQE 423
[3]    RKPPREESSATCVLEQPGALGAQILLVQRPNSGLLAGLWEFPSVTWEPSEQLQRKALLQE 420
[4]    RKPPREESSATCVLEQPGALGAQILLVQRPNSGLLAGLWEFPSVTWEPSEQLQRKALLQE 395

[1]    LQRWAGPLPATHLRHLGEVVHTFSHIKLTYQVYGLALEGQTPVTTVPPGARWLTQEEFHT 469
[2]    LQRWAGPLPATHLRHLGEVVHTFSHIKLTYQVYGLALEGQTPVTTVPPGARWLTQEEFHT 483
[3]    LQRWAGPLPATHLRHLGEVVHTFSHIKLTYQVYGLALEGQTPVTTVPPGARWLTQEEFHT 480
[4]    LQRWAGPLPATHLRHLGEVVHTFSHIKLTYQVYGLALEGQTPVTTVPPGARWLTQEEFHT 455

[1]    AAVSTAMKKVFRVYQGQQPGTCMGSKRSQVSSPCSRKKPRMGQQVLDNFFRSHISTDAHS 529
[2]    AAVSTAMKKVFRVYQGQQPGTCMGSKRSQVSSPCSR------------------------ 519
[3]    AAVSTAMKKVFRVYQGQQPGTCMGSKRSQVSSPCSRKKPRMGQQVLDNFFRSHISTDAHS 540
[4]    AAVSTAMKKVFRVYQGQQPGTCMGSKRSQVSSPCSRKKPRMGQQVLDNFFRSHISTDAHS 515

[1]    LNSAAQ  535
[2]    ------  519
[3]    LNSAAQ  546
[4]    LNSAAQ  521
```

Figure S8. Numeration of MUTYH residues used in the literature.

Numeration after:

[73]. Slupska M.M., Baikalov C., Luther W.M., Chiang J.-H., Wei Y.-F., Miller J.H. (1996) Cloning and sequencing a human homolog (*hMYH*) of the *Escherichia coli mutY* gene whose function is required for the repair of oxidative DNA damage. *J. Bacteriol.*, **178**, p. 3885-3892.

[49]. Trasviña-Arenas C.H., Dissanayake U.C., Tamayo N., Hashemian M., Lin W.-J., Demir M., Hoyos-Gonzalez N., Fisher A.J., Cisneros G.A., Horvath M.P., David S.S. (2025) Structure of human MUTYH and functional profiling of cancer-associated variants reveal an allosteric network between its [4Fe-4S] cluster cofactor and active site required for DNA repair. *Nat. Commun.*, **16:**3596.

[102]. Ohtsubo T., Nishioka K., Imaiso Y., Iwai S., Shimokawa H., Oda H., Fujiwara T., Nakabeppu Y. (2000) Identification of human MutY homolog (hMYH) as a repair enzyme for 2-hydroxyadenine in DNA and detection of multiple forms of hMYH located in nuclei and mitochondria. *Nucleic Acids Res.*, **28**, p. 1355-1364. . https://doi.org/10.1093/nar/28.6.1355.

Isoform $\alpha 1$ in the reference paper, isoform 1 in the GenBank database (NP_036354.1). This is the numeration used in the COSMIC database.
Isoform β3 in the reference paper (same as [3]), isoform 2 in the GenBank database (NP_001041636.2). This is the numeration used in the ClinVar, gnomAD and Million Exome databases.

Highlighted in red are the residues affected by the most frequent founder mutations (Y165C and G382D) responsible for familial adenomatous polyposis type 2 in the populations of European origin, as described in Al-Tassan N., Chmiel N.H., Maynard J., Fleming N., Livingston A.L., Williams G.T., Hodges A.K., Davies D.R., David S.S., Sampson J.R., Cheadle J.P. (2002) Inherited variants of *MYH* associated with somatic G:C→T:A mutations in colorectal tumors. *Nat. Genet.*, **30**, p. 227-232.

Highlighted in blue are the residues for which variants of unknown significance G169D and G202E were studied in this work.

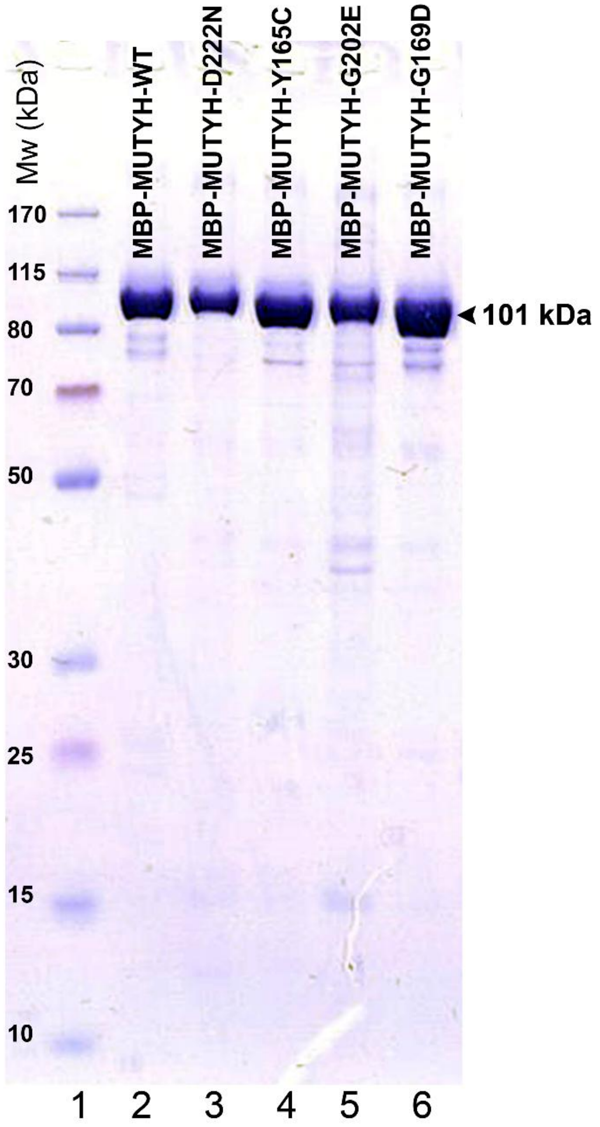

Figure S9. SDS-PAGE of the purified wild-type and mutant variants of MUTYH protein.

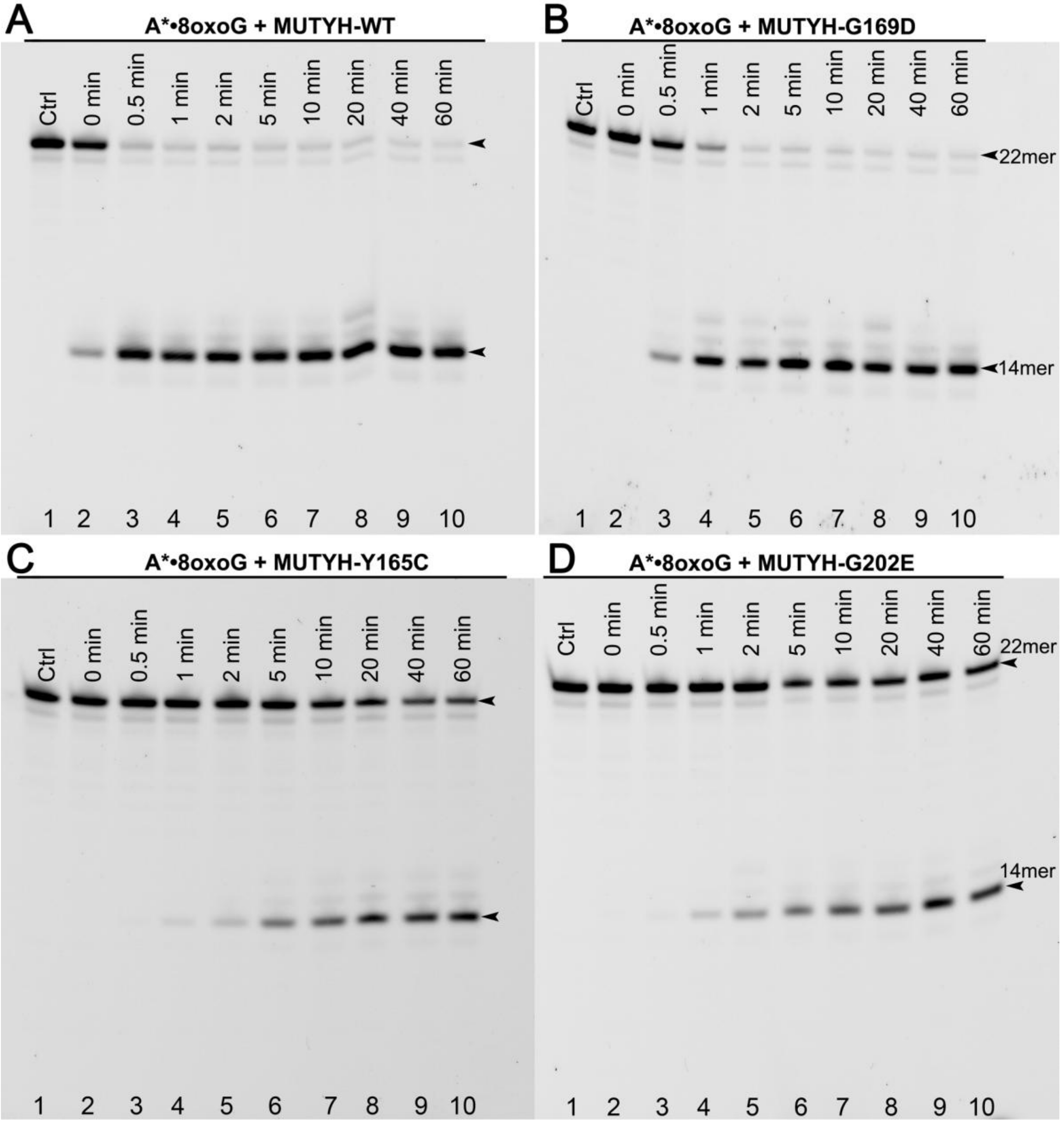

E

22 mer duplex context «DG3», where X is 8oxoG.

^{32}P-d(AGATCAGTCACGAT ***A*** *CGAAGTG)*

(TCTAGTCAGTGCTA ***X*** *GCTTCAC)d*

Figure S10. Time-dependent adenine excision in A•8oxoG oligonucleotide duplex (context "**DG3**") by MUTYH wild-type and mutant variants. (**A-D**) Denaturing PAGE analysis of reaction products. Time courses were performed using 20 nM of 22 mer 5′-Cy5-labelled A*•8oxoG duplex oligonucleotide and 200 nM of MUTYH-WT (**A**), G169D (**B**), Y165C (**C**), and G202E (**D**) proteins for 1h at 37°C. Arrows indicate the sizes of the DNA substrate and cleavage fragments. (**E**) Schematic representation of 22 mer duplex sequence with target adenine residue and opposite modified base. For details see Materials and Methods section.

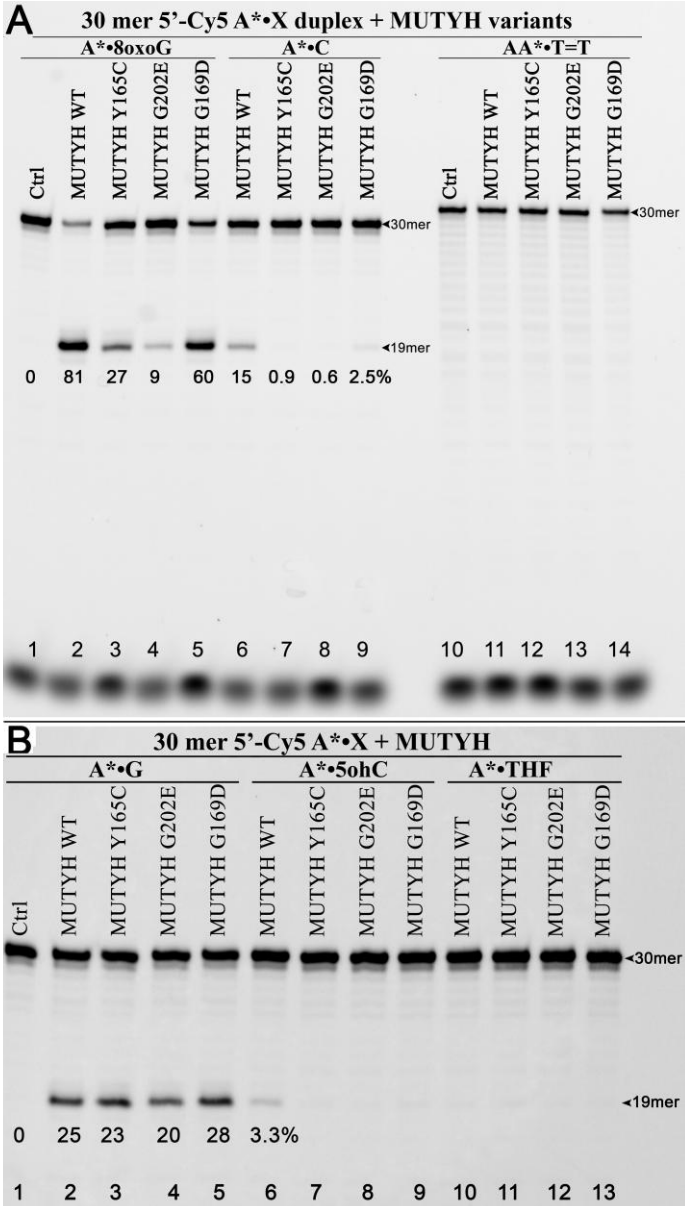

Figure S11. Activities of wild-type and mutant variants of MUTYH towards duplex oligonucleotides containing mismatched adenine opposite various base modifications. (**A, B**) 30 mer A*•X oligonucleotide duplexes (context "**RT**") containing non-bulky base modifications and 30 mer AA*•T=T containing UV pyrimidine dimer. 20 nM of 30 mer 5′-Cy5 labelled A*•8oxoG, A*•G, A*•C, A*•5ohC, A*•THF and AA*•T=T oligonucleotide duplexes were incubated with 200 nM of MUTYH proteins for 1h at 37°C. Arrows indicate the sizes of the DNA substrate and cleavage fragments. The quantities designated above the lane numbers (Panel A: lanes 1-9; Panel B: lanes 1-6 correspond to the percentage of cleavage products. For details, see Materials and Methods section.

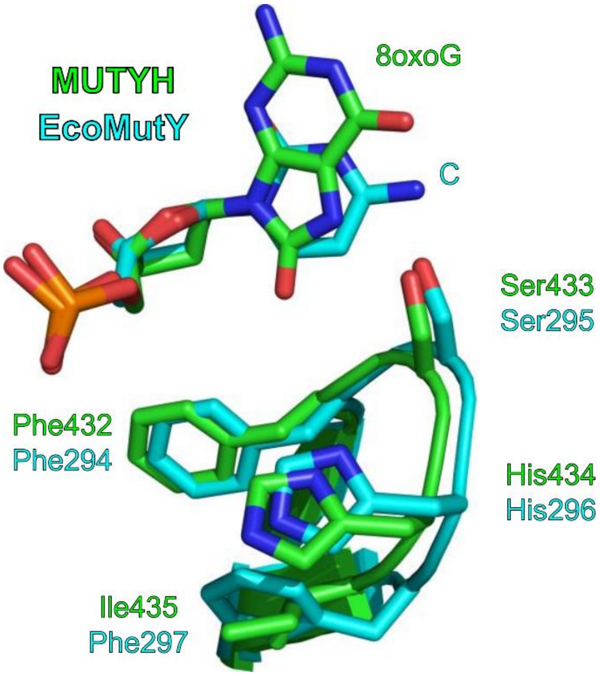

Figure S12. Structure of the tip of the β-hairpin interacting with the orphaned nucleotide in two representative models of MUTYH bound to A•8oxoG-containing DNA (green) and EcoMutY bound to A•C-containing DNA (cyan). Note the movement of the His residue.

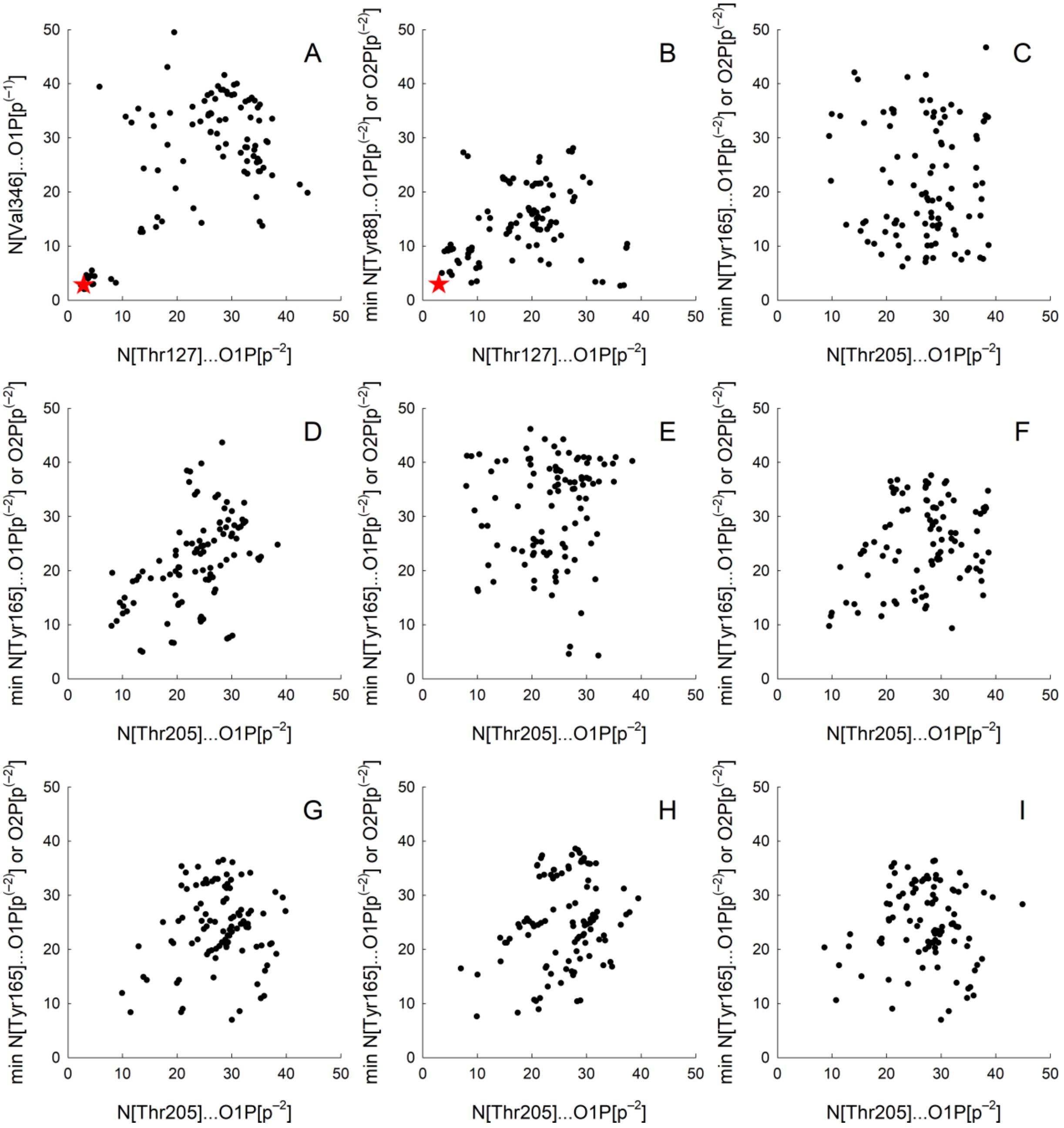

Figure S13. Examples of distributions of 100 highest-scoring docking poses generated by pyDockDNA. (**A**)**,** BstMutY from PDB ID 3G0Q docked on DNA from the same crystal structure (the post-eversion complex). (**B**)**,** BstMutY from 5KN9 docked on DNA from the same crystal structure (the pre-eversion complex). Stars show the distances in the experimental structures. (**C–I**)**,** MUTYH AlphaFold 3 model docked on **(C),** AA•T=T DNA NMR structure (1TTD, orientation calculated for targeting 5′-A opposite 3′-T in the dimer); **(D),** AA•T=T DNA NMR structure (1TTD, orientation calculated for targeting 3′-A opposite 5′-T in the dimer); **(E),** AA•T=T DNA crystal structure with 3′-A flipped out (1VAS). **(F)** CAC•GTG^{Pt} DNA NMR structure (1DA4, model 1); **(G),** CAC•GTG^{Pt} DNA NMR structure (1DA4, model 2); **(H)** CAC•GTG^{Pt} DNA NMR structure (1DA5, model 1); **(I),** CAC•GTG^{Pt} DNA NMR structure (1DA5, model 2).

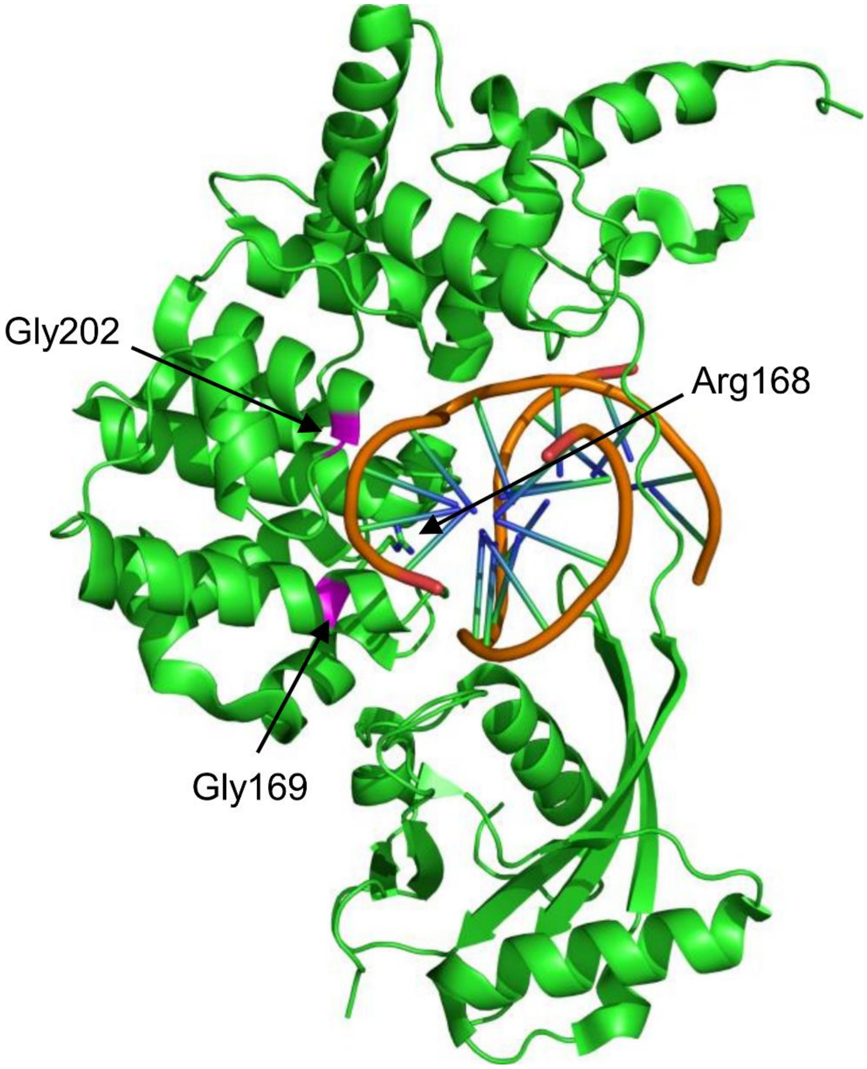

Figure S14. Positions of Gly169 and Gly202 in the MUTYH–DNA structure (PDB ID 8FAY). Arg168 is shown as a stick model.
